# Assessment of Psychosocial and Neonatal Risk Factors for Trajectories of Behavioral Dysregulation Among Young Children From 18 to 72 Months of Age

**DOI:** 10.1001/jamanetworkopen.2023.10059

**Published:** 2023-04-26

**Authors:** Julie A. Hofheimer, Monica McGrath, Rashelle Musci, Guojing Wu, Sarah Polk, Courtney K. Blackwell, Annemarie Stroustrup, Robert D. Annett, Judy Aschner, Brian S. Carter, Jennifer Check, Elisabeth Conradt, Lisa A. Croen, Anne L. Dunlop, Amy J. Elliott, Andrew Law, Leslie D. Leve, Jenae M. Neiderhiser, T. Michael O’Shea, Amy L. Salisbury, Sheela Sathyanarayana, Rachana Singh, Lynne M. Smith, Andréa Aguiar, Jyoti Angal, Hannah Carliner, Cindy McEvoy, Steven J. Ondersma, Barry Lester

**Affiliations:** 1 Department of Pediatrics, University of North Carolina at Chapel Hill, Chapel Hill; 2 Department of Health Sciences, University of North Carolina at Chapel Hill, Chapel Hill; 3 Department of Epidemiology, Johns Hopkins Bloomberg School of Public Health, Baltimore, Maryland; 4 Department of Mental Health, Johns Hopkins Bloomberg School of Public Health, Baltimore, Maryland; 5 Department of Pediatrics, Johns Hopkins University School of Medicine, Baltimore, Maryland; 6 Department of Medical Social Sciences, Feinberg School of Medicine, Northwestern University, Chicago, Illinois; 7 Division of Neonatology, Department of Pediatrics, Cohen Children’s Medical Center at Northwell Health, New Hyde Park, New York; 8 Department of Pediatrics, University of New Mexico Health Sciences Center, Albuquerque; 9 Department of Pediatrics, Albert Einstein College of Medicine, Bronx, New York; 10 Hackensack Meridian School of Medicine, Nutley, New Jersey; 11 Department of Pediatrics, University of Missouri-Kansas City, Children’s Mercy Kansas City, Kansas City; 12 Department of Pediatrics, Wake Forest School of Medicine, Winston-Salem, North Carolina; 13 Department of Psychiatry, Duke University School of Medicine, Durham, North Carolina; 14 Division of Research, Kaiser Permanente Northern California, Oakland, California; 15 Department of Gynecology and Obstetrics, Emory University School of Medicine, Atlanta, Georgia; 16 Avera Research Institute, Sioux Falls, South Dakota; 17 Department of Pediatrics, University of South Dakota School of Medicine, Sioux Falls; 18 Prevention Science Institute, University of Oregon, Eugene; 19 Department of Psychology, Penn State University, State College, Pennsylvania; 20 School of Nursing, Virginia Commonwealth University, Richmond; 21 Department of Pediatrics, University of Washington, Seattle Children’s Research Institute, Seattle; 22 Department of Pediatrics, Tufts University School of Medicine, Boston, Massachusetts; 23 Department of Pediatrics, Harbor-UCLA Medical Center, Torrance, California; 24 Department of Comparative Biosciences, University of Illinois at Urbana-Champaign, Urbana-Champaign; 25 Beckman Institute for Advanced Science and Technology, University of Illinois at Urbana-Champaign, Urbana-Champaign; 26 Department of Psychiatry, Columbia University Vagelos College of Physicians and Surgeons, New York, New York; 27 Department of Pediatrics, Oregon Health & Science University, Doernbecher Children’s Hospital, Portland; 28 Division of Public Health, Michigan State University, East Lansing; 29 Department of Obstetrics, Gynecology, and Reproductive Biology, Michigan State University, East Lansing; 30 Brown Center for the Study of Children at Risk, Women & Infants Hospital, Brown University Alpert School of Medicine, Providence

## Abstract

**Importance:**

Emotional and behavioral dysregulation during early childhood are associated with severe psychiatric, behavioral, and cognitive disorders through adulthood. Identifying the earliest antecedents of persisting emotional and behavioral dysregulation can inform risk detection practices and targeted interventions to promote adaptive developmental trajectories among at-risk children.

**Objective:**

To characterize children’s emotional and behavioral regulation trajectories and examine risk factors associated with persisting dysregulation across early childhood.

**Design, Setting, and Participants:**

This cohort study examined data from 20 United States cohorts participating in Environmental influences on Child Health Outcomes, which included 3934 mother-child pairs (singleton births) from 1990 to 2019. Statistical analysis was performed from January to August 2022.

**Exposures:**

Standardized self-reports and medical data ascertained maternal, child, and environmental characteristics, including prenatal substance exposures, preterm birth, and multiple psychosocial adversities.

**Main Outcomes and Measures:**

Child Behavior Checklist caregiver reports at 18 to 72 months of age, with Dysregulation Profile (CBCL-DP = sum of anxiety/depression, attention, and aggression).

**Results:**

The sample included 3934 mother-child pairs studied at 18 to 72 months. Among the mothers, 718 (18.7%) were Hispanic, 275 (7.2%) were non-Hispanic Asian, 1220 (31.8%) were non-Hispanic Black, 1412 (36.9%) were non-Hispanic White; 3501 (89.7%) were at least 21 years of age at delivery. Among the children, 2093 (53.2%) were male, 1178 of 2143 with Psychosocial Adversity Index [PAI] data (55.0%) experienced multiple psychosocial adversities, 1148 (29.2%) were exposed prenatally to at least 1 psychoactive substance, and 3066 (80.2%) were term-born (≥37 weeks’ gestation). Growth mixture modeling characterized a 3-class CBCL-DP trajectory model: high and increasing (2.3% [n = 89]), borderline and stable (12.3% [n = 479]), and low and decreasing (85.6% [n = 3366]). Children in high and borderline dysregulation trajectories had more prevalent maternal psychological challenges (29.4%-50.0%). Multinomial logistic regression analyses indicated that children born preterm were more likely to be in the high dysregulation trajectory (adjusted odds ratio [aOR], 2.76; 95% CI, 2.08-3.65; *P* < .001) or borderline dysregulation trajectory (aOR, 1.36; 95% CI, 1.06-1.76; *P* = .02) vs low dysregulation trajectory. High vs low dysregulation trajectories were less prevalent for girls compared with boys (aOR, 0.60; 95% CI, 0.36-1.01; *P* = .05) and children with lower PAI (aOR, 1.94; 95% CI, 1.51-2.49; *P* < .001). Combined increases in PAI and prenatal substance exposures were associated with increased odds of high vs borderline dysregulation (aOR, 1.28; 95% CI, 1.08-1.53; *P* = .006) and decreased odds of low vs high dysregulation (aOR, 0.77; 95% CI, 0.64-0.92; *P* = .005).

**Conclusions and Relevance:**

In this cohort study of behavioral dysregulation trajectories, associations were found with early risk factors. These findings may inform screening and diagnostic practices for addressing observed precursors of persisting dysregulation as they emerge among at-risk children.

## Key Points

**Question:**

Can adverse prenatal, neonatal, and psychosocial conditions identify the earliest risk factors associated with persistent behavioral and emotional dysregulation across early childhood?

**Findings:**

In this cohort study of 3934 mother-child pairs, preterm birth and the interactive influences of cumulative prenatal substance exposures and psychosocial adversities were associated with persisting high and borderline Child Behavior Checklist-Dysregulation trajectories across 18 to 72 months of age.

**Meaning:**

These findings suggest salient indicators of resilience and risk for persisting emotional and behavioral dysregulation to inform earlier and more specific behavioral screening and to target interventions with potential to promote adaptive development among at-risk children.

## Teaser

This cohort study investigates children’s emotional and behavioral regulation trajectories and assesses whether psychosocial or neonatal risk factors are associated with persisting dysregulation across early childhood.

## Introduction

Emotional and behavioral dysregulation among school-aged children has an average multinational prevalence of 9% (range, 2%-18%; n = 56 666)^1^ and is associated with subsequently severe behavioral, affective, and cognitive impairments through adulthood.^2,3,4^ Risk factors for children’s persisting emotional and behavioral dysregulation involve the cumulative burden of medical and socioeconomic adversities, as well as parental psychological and substance use challenges.^5,6,7,8,9,10,11^

Children born preterm (<37 weeks’ gestation), and children whose parents have co-occurring psychological and substance use challenges are at elevated risk for persisting dysregulation.^12,13,14,15,16^ Moreover, prematurity, in combination with environmental adversity has been associated with childhood anxiety, attention, mood, and social-communicative disorders that persist into adulthood.^17,18,19^ Preterm children’s outcomes differed from term-born children’s outcomes,^20,21,22^ whereas psychosocial factors were associated with behavioral dysregulation similarly across various samples.^8,23,24^

The Child Behavior Checklist (CBCL/1.5-5)^25^ is a well-validated measure that yields subscores for emotional reactivity, sleep and somatic problems, social withdrawal, anxiety/depression, attention, and aggression.^26^ The latter 3 subscores compose the Dysregulation Profile (CBCL-DP), a global indicator of risk for major impairments throughout adolescence and adulthood.^2,12,20,27,28^ Early dysregulation expressed as difficulties with attention, emotional regulation, and social relationships has been found to persist through childhood,^15,29^ with demonstrated significance identifying subsequent suicidality,^4,30^substance use,^31^ attention deficits, bipolar and major depression, anxiety, and disruptive behavior disorders into adulthood.^3,4^

While prior studies have documented individual medical, economic, and social risk factors for poor behavioral regulation, less is known about the earliest risk factors associated with persisting dysregulation among US children. Thus it is important to examine behavioral trajectories in large, diverse samples with child and caregiver data that includes prenatal and neonatal exposures.

The present study was designed to address gaps regarding the earliest antecedents of persisting behavioral dysregulation across early childhood.^32^ Our goals were to characterize children’s CBCL-DP trajectories and identify early psychosocial and neonatal characteristics associated with resilience compared with persisting dysregulation. We hypothesized that low or improved dysregulation would be associated with more socioenvironmental resources and fewer adversities, and persisting dysregulation would be associated with more adverse perinatal, psychosocial, and environmental exposures. We examined these associations among a diverse multicohort sample of US children enrolled in the National Institutes of Health Environmental influences on Child Health Outcomes (ECHO) program^33,34^ investigation of early-life exposures that affect child health and neurodevelopment.^35^

## Methods

### Participants

Participants included 3934 children aged 18 to 72 months born in 1990 to 2019 and enrolled in 20 ECHO cohorts (eTable 1 in Supplement 1). Inclusion criteria were: (1) singleton births; (2) data on prenatal exposure to alcohol, nicotine, marijuana, opioids, and illicit substances; and (3) CBCL/1.5-5 data^25^ at ages 18 through 72 months.

For each local cohort and for ECHO, data use agreements were approved by their respective institutional review boards and written informed consent for maternal and child participation were obtained. This cohort study followed the Strengthening the Reporting of Observational Studies in Epidemiology (STROBE) reporting guideline.

### Procedures

#### Prenatal Substance Exposure

Prenatal substance use data (eFigure 1 in Supplement 1) was obtained from self-report, medical record abstraction, and/or biological assays^36,37,38^: (1) nicotine (tobacco; nicotine patch, gum, lozenges, inhaler); (2) alcohol; (3) marijuana; (4) opioids (morphine, codeine, Percodan, OxyContin, fentanyl, heroin); and (5) illicit substances (cocaine, methamphetamines, hallucinogens, inhalants; specified unprescribed or misused pharmaceutical). To quantify cumulative prenatal exposures, each category endorsed was assigned a value of 1 and the sum of each substance category used was calculated^39,40^ (range: 0-5).

#### Socioenvironmental Characteristics

Maternal education was categorized as (1) less than high school degree, (2) high school graduate or GED, (3) some college, and (4) Bachelor’s degree and above. Self-identified race and ethnicity were categorized as Hispanic, non-Hispanic American Indian or Alaska Native, non-Hispanic Asian, non-Hispanic Black, non-Hispanic Native Hawaiian or Pacific Islander, non-Hispanic White, non-Hispanic other race or ethnicity, and non-Hispanic multiple races. Race and ethnicity were collected by self-report to describe the diversity among ECHO mothers and children. Marital or partner status was defined as married or living with a partner, or not married nor partnered. Health insurance type was dichotomized as none and publicly subsidized, or private and employer-provided insurance.

#### Psychological History

Maternal psychological history was operationalized using data on psychiatric diagnoses and depressive symptom scores on self-reported questionnaires and/or medical records. Maternal psychological history was defined as yes if there was either a prior psychiatric diagnosis or Patient-Reported Outcomes Measurement Information System v1.0 (PROMIS)^41^ depressive symptom T-score greater than or equal to 55 prior to the first CBCL assessment.

Maternal psychiatric diagnoses included major depression, dysthymia, phobias, and bipolar, anxiety, panic, obsessive-compulsive, posttraumatic stress, and attention-deficit disorders. Depressive symptoms were reported on the following standardized screening measures: (1) PROMIS v1.0^42,43^; (2) Edinburgh Postnatal Depression Scale^44^; (3) Adult Self-Report Achenbach System Depression Problems Syndrome Scale; (4) Brief Symptom Inventory; (5) Center for Epidemiological Studies Depression Scale; (6) Patient Health Questionnaire; (7) Beck Depression Inventory^45^; and (8) Kessler 6 Mental Health Scale.^46,47^ Measures were harmonized using PROsetta Stone, which incorporates item response theory and equipercentile score–linking with the PROMIS Depression T-score metric (mean [SD]: 50 [10])^44^ in cross-walk conversion tables. Validated PROMIS Depression thresholds have identified T-scores greater than or equal to 55 as a positive screen.^41,48^

#### Psychosocial Adversity Index

We created a Psychosocial Adversity Index (PAI) to quantify the cumulative burden of adverse conditions in which children were developing, assigning a value of 1 for each for the following criteria based on previously validated methods^23,49^: (1) maternal age at delivery less than 21 years; (2) maternal education less than some college (based on postrecession challenges for adults who did not attend college^9,50^); (3) publicly subsidized or no health insurance; (4) single-parent household (unmarried or not living with a partner); and (5) prior maternal psychiatric diagnosis or positive depressive symptom screen. We modeled PAI (range: 0-5) as a continuous variable.

#### Birth Outcomes

Gestational age (GA) groups included term (≥37 weeks), preterm (<37 weeks), moderate or late preterm (32-36 weeks), and very preterm (<32 weeks). Small-for-gestational-age (SGA; <10th percentile) and large-for-gestational-age (LGA; >90th percentile) were defined using the International Fetal and Newborn Growth Consortium for the 21st Century fetal growth standards.^51^ Postnatal length of hospital stay (LOS) was calculated from dates of birth and neonatal discharge. GA-specific median LOS was determined (when continuous LOS was available); children were categorized as less than vs greater than or equal to the median GA-specific LOS as a proxy for illness severity at birth.

#### Child Behavior Checklist/1.5-5

Across cohort-specific 18- to 72-month assessment protocols, caregivers completed CBCLs per standardized reporting procedures,^25^ with items rated: 0 = not true (as far as you know), 1 = somewhat or sometimes true, and 2 = very or often true. Subscores were calculated for emotional reactivity, sleep and somatic problems, withdrawn, anxiety/depression, attention, and aggression, and transformed into standardized T-scores (mean [SD]: 50 [10]).^25^ The CBCL-DP is the sum of subscores for *Anxious/Depressed* (8 items), *Attention Problems* (5 items), and *Aggressive Behavior Problems* (19 items); scores greater than 180 defined clinically validated dysregulation using categories validated in preschool samples.^4,52^

### Statistical Analysis

Univariate differences among maternal and child characteristics associated with CBCL outcomes were examined using χ^2^ and *t* tests for categorical and continuous variables, respectively. Univariate and multivariable significance was set at 2-sided *P* < .05. We used growth mixture modeling (GMM) to identify mutually exclusive subpopulations’ patterns of change in dysregulation from ages 18 to 72 months. Age epochs for repeated measures were 18 to 35 months, 36 to 47 months, 48 to 59 months, and 60 to 72 months. If more than 1 CBCL was available in an individual age epoch, their mean CBCL for the age epoch was calculated.

GMM, a latent variable modeling approach, estimates a categorical latent variable (class), which allows for different groups of individual growth trajectories to vary around class-specific means using repeated measures of CBCL dysregulation.^53,54,55^ The resulting model created a single latent class, as well as class-specific latent intercepts and latent slopes. Beginning first with a single latent class, intercept, and slope, systematic iteration through models estimated increasing numbers of classes. Bayesian Information Criteria (BIC), Lo-Mendell-Rubin adjusted likelihood ratio test (LMR-LRT), and substantive interpretation identified the best-fitting model. Covariates to be used in the latent regression were included as auxiliary variables, and a new data set with posterior class probabilities, modal class assignment, and auxiliary variables specified.

Second in a 3-step approach examined the association of covariates with latent classes to account for measurement error in latent classification. This new data set was used to perform step 3: multinomial logistic regression with children nested in cohorts and adjusted for children’s birth sex and year investigated trajectory class membership associations with cumulative prenatal substance exposure (continuous), PAI (continuous), and GA group (term vs preterm) to calculate odds ratios and 95% CIs. Standard errors were adjusted for clustering at the cohort level via sandwich estimators. We assessed effect modification by including interaction terms for prenatal substance exposure with PAI, and prenatal substance exposure with GA group.

In this study, missing CBCL and covariate data reflected different cohort protocol content and ages at assessment. Multivariable analyses adjusted for cohort membership to account for these differences, and missing data was handled in 2 separate ways. During the class enumeration step, the standard full information maximum likelihood procedure^56,57,58^ was used for participants missing CBCL-DP data for any age epoch. The full maximum likelihood procedure involves the estimation of population parameters by determining the value that maximizes the likelihood function based on the sample data included. To handle missing covariate data, multiple imputation was used based on current standards^54^ for data treated as missing at random when the missing data are unrelated to the outcome of interest (ie, CBCL-DP trajectory). Multiple imputation in Mplus used Markov Chain Monte Carlo methods. All data management and analysis were performed in R statistical software version 4.1.0 (R Project for Statistical Computing, including tidyverse, data.table, mice) and Mplus version 8.8^59^ (including MplusAutomation) from January to August 2022.

## Results

**Figure 1.**
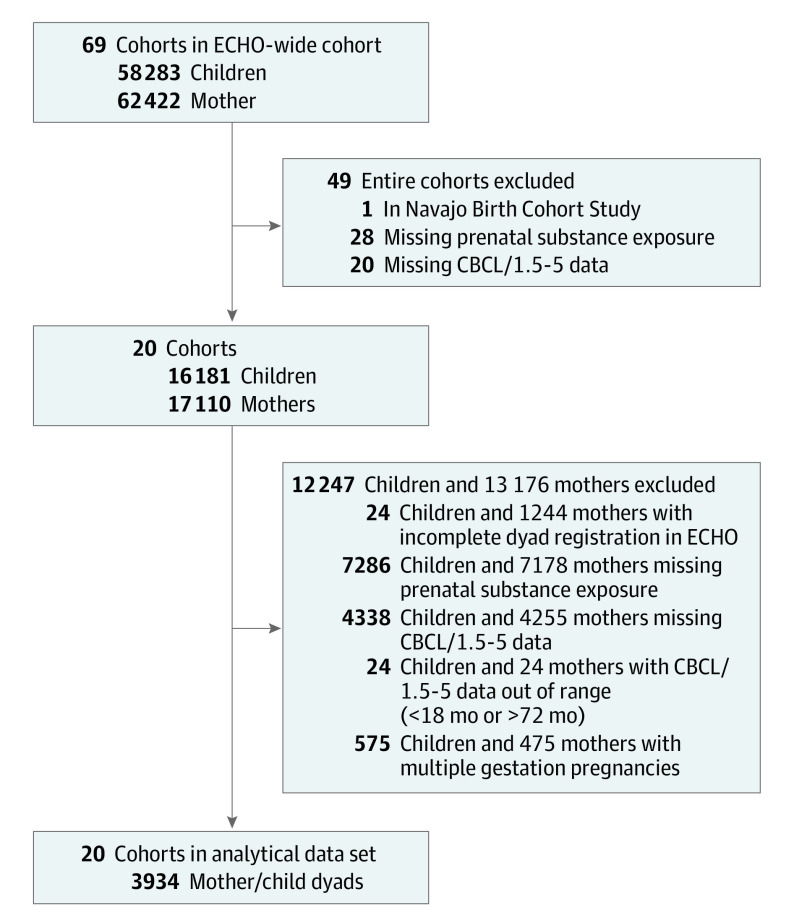
Flowchart for Eligibility and Selection of the Multicohort Sample of Study Participants CBCL/1.5-5 indicates Child Behavior Checklist; ECHO, National Institutes of Health Environmental influences on Child Health Outcomes program.

There were 3934 mother-child pairs from 20 cohorts (eFigure 2, eTable 1 in Supplement 1) that met the inclusion criteria (Figure 1). Participant characteristics are summarized in Table 1. Among 3934 mothers, 718 (18.7%) self-identified as Hispanic, 252 (7.2%) as non-Hispanic Asian, 1220 (31.8%) as non-Hispanic Black, 12 (<0.1%) as non-Hispanic Native Hawaiian or Pacific Islander, 1412 (36.9%) as non-Hispanic White, 5 (<.01%) as non-Hispanic other races, and 165 (4.3%) as non-Hispanic multiple races. Overall, 3073 mothers (84.0%) had some college or above; 3501 mothers (89.7%) were at least 21 years of age at delivery; 28.0% (493 of 1759) were neither married nor living with a partner; 60.8% (1303 of 2143) had publicly subsidized or no health insurance. Approximately one-fourth of mothers had a prior psychiatric diagnosis (699 mothers [22.1%]) or positive depressive screen (664 mothers [27.1%]), and 1178 of 2143 with data (55.0%) were in the multirisk PAI group (PAI score = 2-5; eTable 4 in Supplement 1 presents differences in PAI risk factors).

**Table 1.** ECHO Multicohort Sample Characteristics

Characteristics	Study sample, No. (%)
No. of mother-child pairs	3934 (100.0)
Maternal characteristics	
Age at delivery, No. (%) with data	3903 (99.2)
<21 y	402 (10.3)
≥21 y	3501 (89.7)
Race and ethnicity, No. (%) with data	3831 (97.4)
Hispanic any race	718 (18.7)
Non-Hispanic American Indian or Alaska Native	24 (<1)
Non-Hispanic Asian	275 (7.2)
Non-Hispanic Black	1220 (31.8)
Non-Hispanic Native Hawaiian or Pacific Islander	12 (<1)
Non-Hispanic White	1412 (36.9)
Non-Hispanic self-reported other race	5 (<1)
Non-Hispanic multiple race	165 (4.3)
Highest level of education, No. (%) with data	3657 (93.0)
High school or less	584 (16.0)
Some college and above	3073 (84.0)
Some college	1158(31.7)
Bachelor’s degree and above	1915 (52.4)
Marital/partner status, No. (%) with data	1759 (44.7)
Married or living with a partner	1266 (72.0)
Public/no insurance, No. (%) with data	2143 (54.5)
Yes	1303 (60.8)
History of maternal psychiatric disorder, No. (%) with data	3158 (80.3)
Yes	699 (22.1)
Maternal PROMIS depressive symptom score, No. (%) with data	2451 (62.3)
T-score, mean (min-max) [SD]	50 (33-78) [8.3]
T-score ≥55	664 (27.1)
Psychosocial Adversity Index (PAI), No. (%) with data	2143 (54.5)
PAI, mean (min-max) [SD]	1.7 (0-5) [1.2]
PAI = 0	482 (22.5)
PAI = 1	483 (22.5)
PAI = 2	589 (27.5)
PAI = 3	432 (20.2)
PAI = 4	141 (6.6)
PAI = 5	16 (<1)
Low risk (0-1)	965 (45.0)
Multirisk (2-5)	1178 (55.0)
Substance use during pregnancy, No. (%) with data	3934 (100.0)
Any substance use during pregnancy = yes	1148 (29.2)
No. of substances used, mean (min-max, SD)	0.52 (0-5, 0.86)
Alcohol	670 (17.0)
Nicotine	478 (12.2)
Marijuana	290 (7.4)
Illicit substances^a^	96 (2.4)
Any opioids	66 (1.7)
Child characteristics	
Sex of child, No. (%) with data	3934 (100.0)
Male	2093 (53.2)
Female	1841 (46.8)
Race and ethnicity, No. (%) with data	3838 (97.6)
Hispanic any race	899 (23.4)
Non-Hispanic American Indian or Alaska Native	20 (<1)
Non-Hispanic Asian	190 (5.0)
Non-Hispanic Black	1156 (30.1)
Non-Hispanic Native Hawaiian or Pacific Islander	6 (<1)
Non-Hispanic White	1218 (31.7)
Non-Hispanic self-reported other race	5 (<1)
Non-Hispanic multiple race	344 (9.0)
Calendar year of childbirth, No. (%) with data	3934 (100.0)
Before 2005	184 (4.7)
2005-2010	1096 (27.9)
2011-2015	1765 (44.9)
2016-2019	889 (22.6)
Gestational age in weeks, No. (%) with data	3825 (97.2)
Mean (min-max) [SD]	37 (22-43) [4.4]
Term (≥37 weeks)	3066 (80.2)
Preterm (<37 weeks)	759 (19.8)
Moderate or late preterm (32-36 weeks)	243 (6.4)
Very preterm (22-31 weeks)	516 (13.5)
Large or small for gestational age, No. (%) with data	3762 (95.6)
Small for gestational age	301 (8.0)
Large for gestational age	471 (12.5)
Head circumference at delivery in cm, No. (%) with data	3135 (79.7)
Mean (min-max) [SD]	32 (18-40) [4]
APGAR scores at 1 min after birth, No. (%) with data	2989 (76.0)
Mean (min-max) [SD]	7.5 (0-10) [2]
APGAR scores at 5 min after birth, No. (%) with data	3000 (76.3)
Mean (min-max) [SD]	8.5 (0-10) [1.3]
Length of hospital stay, No. (%) with data	3142 (79.9)
Mean (min-max) [SD]	36 (0-418) [51]
<1 day	76 (2.4)
≥1 and ≤4 days	2384 (75.9)
>5 days	682 (21.7)

Abbreviations: APGAR, Appearance, Pulse, Grimace, Activity, and Respiration test; max, maximum; min, minimum; PAI, Psychosocial Adversity Index; PROMIS, Patient-Reported Outcomes Measurement Information System.

^a^ Illicit substances: cocaine, heroin, methamphetamines, hallucinogens, inhalants, or any specified use of unprescribed or misused pharmaceutical (eg, amphetamines, benzodiazepines, ketamine).

Among 3934 children, 2093 (53.2%) were male; 3066 (80.2%) were term-born (≥37 weeks); 243 (6.4%) were moderate/late preterm (32-36 weeks); 516 (13.5%) were very preterm (<32 weeks); and 2990 (79.5%) had appropriate birthweight for gestational age. Child and maternal race and ethnicity frequencies were similar to each other, with more children reported as multiple races. Patterns of prenatal substance exposures (eFigure 1 in Supplement 1) indicated 1148 (29.2%) were exposed to at least 1 substance: alcohol (17.0%), nicotine (12.2%), marijuana (7.4%), opioids (1.7%), and illicit substances (2.4%); 23.6% were exposed to 2 or more substances.

### CBCL-DP Trajectories

Among 7516 CBCLs for 3934 children, 1930 children had 1 CBCL; 1267 children had 2 CBCLs; 399 children had 3 CBCLs; and 338 children had 4 or more CBCLs due to differences in cohort-specific protocols.

**Figure 2.**
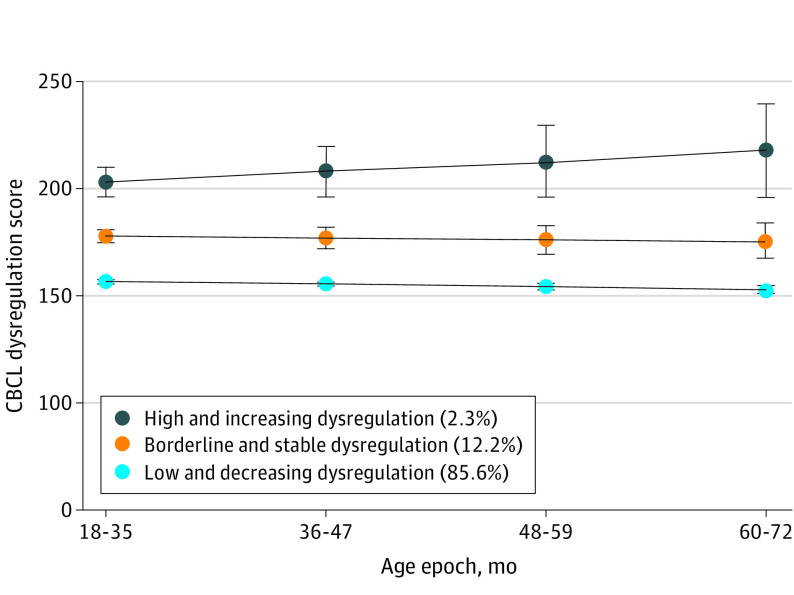
Growth Mixture Modeling Results: Trajectories of Child Behavior Checklist (CBCL)-Dysregulation Profiles From 18 to 72 Months of Age Test statistics for the 3-class model in eTable 2 are available in Supplement 1. A total of 7516 CBCL-Preschool parent-reports were obtained among 3934 children; 1930 children had 1 CBCL record; 1267 children had 2 CBCL records; 399 children had 3 CBCL records; and 338 children had 4 or more CBCL records. Error bands represent 95% CIs.

GMM procedures characterized CBCL-DP trajectories across age epochs, where BIC and LMR-LRT (eTable 2 in Supplement 1) indicated a 3-class solution fit the data best. Three-class model entropy was 0.92; mean posterior class probabilities ranged from 0.89 to 0.95. Figure 2 illustrates CBCL-DP trajectory classes: (1) high and increasing dysregulation (n = 89; 2.3%); (2) borderline and stable dysregulation (n = 479; 12.3%); and (3) low and decreasing dysregulation (n = 3366; 85.6%).

#### CBCL-DP and Subscores Among Trajectory Classes

 Overall, 10.3% (n = 405) of CBCL-DP scores were in the clinical range (mean [range], 158.6 [150-262]). Table 2 indicates that children in the high and borderline dysregulation trajectories also had higher subscores for all 3 CBCL-DP components (attention, anxiety/depression, and aggression), and scores in the clinical range for emotional reactivity, withdrawn, somatic, and sleep problems on at least 1 assessment across 18 to 72 months of age.

**Table 2.** Maternal and Child Characteristics Among Child Behavior Checklist Dysregulation Profile Trajectory Classes

Characteristics	No. (%)			*P* value
	Class 1: High and increasing DP	Class 2: Borderline and stable DP	Class 3: Low and decreasing DP	
No. of mother-child pairs	89 (2.3)	479 (12.2)	3366 (85.6)	
Maternal characteristics				
Age at delivery, No. (%) with data	88 (99.0)	470 (98.1)	3345 (99.0)	
<21 y	11 (12.5)	59 (12.6)	332 (9.9)	.83
≥21 y	77 (87.5)	411 (87.4)	3013 (90.1)	
Highest level of education, No. (%) with data	83 (93.3)	437 (91.2)	3137 (93.2)	
High school or less	26 (31.3)	116 (26.5)	442 (14.1)	<.001
Some college and above	57 (68.7)	321 (73.5)	2695 (85.9)	
Marital/partner status, No. (%) with data	49 (55.1)	235 (49.1)	1475 (43.8)	
Married or living with a partner	23 (46.9)	154 (65.5)	1089 (73.8)	<.001
Public/no insurance, No. (%) with data	58 (65.2)	296 (61.8)	1789 (53.1)	
Yes	51 (87.9)	215 (72.6)	1037 (58.0)	<.001
History of maternal psychiatric disorder, No. (%) with data	64 (71.9)	381 (79.5)	2713 (80.6)	
Yes	32 (50.0)	112 (29.4)	555 (20.5)	<.001
Maternal PROMIS depressive symptom score, No. (%) with data	64 (71.9)	336 (70.1)	2051 (60.9)	
Mean (min-max) [SD]	52 (39-76) [8.7]	53 (33-78) [8.8]	50 (33-76) [8.1]	<.001
T-score ≥55	25 (39.1)	139 (41.4)	500 (24.4)	<.001
Psychosocial Adversity Index score, No. (%) with data	58 (65.2)	296 (61.8)	1789 (53.1)	
Mean (min-max) [SD]	2.8 (1-5) [1]	2.1 (0-5) [1.2]	1.6 (0-5) [1.2]	<.001
Low (0-1)	8 (13.8)	85 (28.7)	872 (48.8)	<.001
Multirisk (2-5)	50 (86.2)	211 (71.3)	917 (51.3)	
Substance use during pregnancy, No. (%) with data	89 (100.0)	479 (100.0)	3366 (100.0)	
Any substance use during pregnancy = yes	34 (38.2)	142 (29.6)	972 (28.9)	.93
No. of substances used, mean (min-max) [SD]	0.51 (0-4) [0.82]	0.53 (0-4) [0.87]	0.52 (0-5) [0.86]	.90
Alcohol	14 (15.7)	59 (12.3)	597 (17.7)	<.001
Nicotine	21 (23.6)	75 (15.7)	382 (11.3)	.01
Marijuana	6 (6.7)	43 (9.0)	241 (7.2)	.13
Illicit substances^a^	<5	<20 (<5)	77 (2.3)	.28
Any opioids	<5	<15 (<5)	50 (1.5)	.04
Child characteristics				
Sex of child assigned at birth, No. (%) with data	89 (100.0)	479 (100.0)	3366 (100.0)	
Male	57 (64.0)	276 (57.6)	1760 (52.3)	<.001
Female	32 (36.0)	203 (42.4)	1606 (47.7)	
Gestational age (GA) in weeks, No. (%) with data	87 (100)	471 (100)	3267 (100)	
Term (≥37 wk)	48 (55.2)	346 (73.5)	2672 (81.8)	<.001
Preterm (<37 wk)	39 (44.8)	125 (26.5)	595 (18.2)	
Very preterm (22-31 wk)	32 (36.8)	94 (20.0)	390 (11.9)	
Moderate/late preterm (32-36 wk)	7 (8.0)	31 (76.6)	205 (6.3)	
Length of hospital stay, No. (%) with data	73 (82.0)	377 (78.7)	2692 (80.0)	
<1 d	0	6 (1.6)	70 (2.6)	NA
≥1 d to ≤4 d	34 (46.6)	267 (70.8)	2083 (77.4)	NA
>5 d	39 (53.4)	104 (27.6)	539 (20.0)	NA
Mean (min-max) [SD], d	65 (1-183) [52]	52 (0-269) [55]	32 (0-418) [49]	<.001
Length of hospital stay, No. (%) with data	48 (53.9)	222 (46.3)	1491 (44.3)	NA
≥37 wk	16 (33.3)	126 (56.8)	1033 (69.3)	
<median	5 (31.3)	26 (20.6)	173 (16.8)	.12
>median	11 (68.8)	100 (79.4)	860 (83.3)	
32 < GA < 36 wk	<5	<10 (<5)	88 (5.9)	
<median	0	5	35 (39.8)	.001
>median	<5	<5	53 (60.2)	
22 < GA < 32 wk	30 (62.5)	89 (40.1)	370 (24.8)	
<median	8 (26.7)	45 (50.6)	175 (47.3)	.004
>median	22 (73.3)	44 (49.4)	195 (52.7)	
Child Behavior Checklist/1.5-5 y measures^b^				
Dysregulation Profile, No. (%) with data	89 (100.0)	479 (100.0)	3366 (100.0)	
Dysregulation Profile score mean (min-max) [SD]	205 (150-256) [21]	176 (150-262) [13]	155 (150-213) [6.8]	NA
Dysregulation Profile score in the clinical range >180, No. (%)	89 (100.0)	260 (54.3)	56 (1.7)	NA
Child Behavior Checklist/1.5-5 y Symptom Subscores				
Anxious/depressed, No. (%) with data	89 (100.0)	479 (100.0)	3366 (100.0)	
Mean (min-max) [SD]	63 (50-96) [9.7]	56 (50-96) [6.4]	51 (50-70) [2.8]	NA
Borderline or clinical range, No. (%)	54 (60.7)	102 (21.3)	41 (1.2)	NA
Attention problems, No. (%) with data	89 (100.0)	479 (100.0)	3366 (100.0)	
Mean (min-max) [SD]	69 (50-80) [7.6]	61 (50-80) [7.1]	52 (50-77) [3.8]	NA
Borderline or clinical range, No. (%)	77 (86.5)	218 (45.5)	133 (4.0)	NA
Aggressive problems, No. (%) with data	89 (100.0)	479 (100.0)	3366 (100.0)	
Mean (min-max) [SD]	73 (50-100) [12]	59 (50-91) [6.8]	51 (50-93) [3]	NA
Borderline or clinical range, No. (%)	78 (87.6)	152 (31.7)	38 (1.1)	NA
Emotionally reactive, No. (%) with data	89 (100.0)	479 (100.0)	3366 (100.0)	
Mean (min-max) [SD]	67 (50-97) [10]	58 (50-93) [7]	52 (50-83) [3.8]	NA
Borderline or clinical range, No. (%)	75 (84.3)	173 (36.1)	138 (4.1)	NA
Somatic complaints, No. (%) with data	89 (100.0)	479 (100.0)	3366 (100.0)	
Mean (min-max) [SD]	59 (50-92) [9.3]	55 (50-84) [6.6]	52 (50-78) [4.4]	NA
Borderline or clinical range, No. (%)	35 (39.3)	103 (21.5)	219 (6.5)	NA
Withdrawn, No. (%) with data	89 (100.0)	479 (100.0)	3366 (100.0)	
Mean (min-max) [SD]	67 (50-91) [11]	59 (50-94) [8]	53 (50-88) [4.8]	NA
Borderline or clinical range, No. (%)	57 (64.0)	155 (32.4)	183 (5.4)	NA
Sleep problems, No. (%) with data	89 (100.0)	479 (100.0)	3366 (100.0)	
Mean (min-max) [SD]	63 (50-100) [12]	57 (50-94) [7.4]	53 (50-88) [4.6]	NA
Borderline or clinical range, No. (%)	40 (44.9)	76 (15.9)	130 (3.9)	NA

Abbreviations: DP, dysregulation profile; max, maximum; min, minimum; NA, not applicable.

^a^ Illicit substances: cocaine, heroin, methamphetamines, hallucinogens, inhalants, or any specified use of unprescribed or misused pharmaceutical (eg, amphetamines, benzodiazepines, ketamine).

^b^ If more than 1 CBCL was available within age epochs, all available information was used, and scores were averaged within epochs.

#### Maternal and Child Characteristics Among CBCL-DP Trajectory Classes

High and borderline dysregulation was more prevalent among boys vs girls, and among preterm vs term-born children (Table 2). The high dysregulation class had the highest exposure to at least 1 substance (38.2% [n = 34]) and to nicotine (23.6% [n = 21]). Alcohol exposure was highest in the low dysregulation class; patterns of marijuana, opioid, and illicit substance exposure were similar among classes.

Mean (SD) PAI scores were highest in the high dysregulation class (2.8 [1]) compared with the borderline (2.1 [1.2]) and low (1.6 [1.2]) dysregulation classes (*P* < .001). Compared with high and borderline dysregulation classes, children in the low dysregulation class were more likely to have mothers who had some college education, private health insurance, and were married or living with a partner. Compared with children in the low dysregulation class, children in the high and borderline dysregulation classes had more prevalent maternal psychiatric diagnoses and positive depression screens. Furthermore, prior psychiatric diagnoses ranged from 19.4% to 50.0% among mothers who used 1 psychoactive substance during pregnancy compared with 16.1% among mothers who did not use any substance (eTable 3 in Supplement 1).

#### Multivariable Associations with Dysregulation Trajectory Classes

Multinomial logistic regression results (Table 3) for whether girls were less likely than boys to be in the high and borderline vs the low dysregulation trajectory were not statistically significant (high vs low: adjusted odds ratio [aOR], 0.60; 95% CI 0.36-1.01; *P* = .05; borderline vs low: aOR, 0.78; 95% CI, 0.59-1.03; *P* = .09). Compared with the low dysregulation trajectory, preterm children were more likely to be in the high (high vs low: aOR, 2.76; 95% CI, 2.08-3.65; *P* < .001) and borderline (borderline vs low: aOR, 1.36; 95% CI, 1.06-1.76; *P* = .02) dysregulation trajectory and less likely to be in the high vs borderline trajectory (aOR, 0.49; 95% CI, 0.36-0.68; *P* < .001). Increasing PAI was associated with membership in the high (aOR, 1.94; 95% CI, 1.51-2.49; *P* < .001) and borderline (aOR, 1.45; 95% CI, 1.32-1.60; *P* < .001), as well as lower odds of membership in the borderline vs high dysregulation trajectory (aOR, 0.49; 95% CI, 0.36-0.68; *P* < .001).

**Table 3.** Multivariable Analyses of Child Behavior Checklist-DP Trajectory Classes^a^

Variable	Class 2 on class 1: borderline and stable DP vs high and increasing DP		Class 1 on class 3: high and increasing DP vs low and decreasing DP		Class 2 on class 3: borderline and stable DP vs low and decreasing DP	
	OR (95% CI)	*P* value	OR (95% CI)	*P* value	OR (95% CI)	*P* value
Model 1						
Female child sex	1.30 (0.78-2.18)	.32	0.60 (0.36-1.01)	.05	0.78 (0.59-1.03)	.09
Prenatal substance exposure	0.95 (0.71-1.26)	.71	1.1 (0.93-1.3)	.29	1.04 (0.84-1.29)	.73
PAI	0.75 (0.6-0.94)	.01	1.94 (1.51-2.49)	<.001	1.45 (1.32-1.6)	<.001
Preterm birth	0.49 (0.36-0.68)	<.001	2.76 (2.08-3.65)	<.001	1.36 (1.06-1.76)	.02
Model 2						
Female child sex	1.28 (0.77-2.12)	.34	0.61 (0.37-1.01)	.06	0.78 (0.59-1.04)	.09
Prenatal substance exposure	0.44 (0.24-0.8)	.007	2.43 (1.41-4.19)	.001	1.07 (0.63-1.81)	.79
PAI	0.67 (0.55-0.81)	<.001	2.19 (1.75-2.75)	<.001	1.46 (1.32-1.62)	<.01
Preterm birth	0.49 (0.36-0.67)	<.001	2.78 (2.11-3.65)	<.001	1.36 (1.05-1.77)	.02
PAI × prenatal substance exposure	1.28 (1.08-1.53)	.006	0.77 (0.64-0.92)	.005	0.99 (0.86-1.13)	.84
Model 3						
Female child sex	1.30 (0.78-2.18)	.31	0.60 (0.36-1.01)	.05	0.78 (0.59-1.03)	.09
Prenatal substance exposure	0.92 (0.55-1.56)	.77	1.10 (0.83-1.46)	.50	1.02 (0.76-1.36)	.89
PAI	0.75 (0.6-0.94)	.01	1.94 (1.51-2.49)	<.001	1.45 (1.32-1.6)	<.001
Preterm birth	0.48 (0.37-0.62)	<.001	2.77 (2.12-3.63)	<.001	1.33 (1.03-1.72)	.03
Preterm birth × prenatal substance use	1.07 (0.58-1.97)	.84	0.99 (0.68-1.46)	.97	1.06 (0.81-1.39)	.67

Abbreviations: DP, dysregulation profile; OR, odds ratio; PAI, Psychosocial Adversity Index.

^a^ Adjusted ORs (95% CI) are displayed for the associations between prenatal substance use (continuous), PAI (continuous), and GA group at birth (term vs preterm birth) with membership in each trajectory group. Interactions terms are also included. Analyses nested children in cohorts and adjusted for child sex (reference = male sex) and child birth year; sample and cell sizes did not permit the simultaneous testing of multiple interaction terms.

Prenatal substance exposure was not significantly associated with dysregulation trajectory class. However, increasing PAI coupled with increasing prenatal substance exposure was associated with increased odds of membership in the high vs borderline trajectory (aOR, 1.28; 95% CI, 1.08-1.53; *P* = .006) and decreased odds of membership in the low trajectory (aOR, 0.77; 95% CI, 0.64-0.92; *P* = .005). Dysregulation trajectories were not significantly associated with interactions involving GA group.

## Discussion

In this large, diverse US sample, we identified early and potentially modifiable risk factors associated with persisting behavioral and emotional dysregulation, including preterm birth and combined prenatal substance exposures and psychosocial adversities. Among children born preterm, increasing lengthier neonatal hospitalization, an indicator of more severe illness, was associated with persisting dysregulation.

Extending prior work, behavioral and emotional resilience was evident in 85.6% of children who exhibited low and decreasing dysregulation. Importantly, half of these children were developing in families facing multiple psychosocial adversities, and 1.2% to 6.5% had various CBCL subscores in the borderline or clinical range (Table 2). Despite their multiple challenges, these children were found to have low and decreasing dysregulation compared with children with similar risk exposures in the high and borderline trajectories.

Additional risk for persisting dysregulation was evident in the 54.3% of children in the borderline dysregulation trajectory, whose scores were in the clinical range and the maximum score exceeded the high dysregulation class maximum. These scores suggest the importance of early behavioral screening and diagnostic services to identify extremes in specific domains for targeting interventions. Clinical referral thresholds were indicated by CBCL-DP T-scores greater than 180 and T-scores greater than 65 for emotional reactivity, withdrawn, somatic, and sleep problems on at least 1 assessment, reflecting potentially modifiable precursors of persisting dysregulation.^60,61^

In this study, CBCL-DP associations with prematurity are comparable to prior studies using similar measures related to behavioral problems at 6 to 19 years.^20,22,62^ Across GA groups, our results are also consistent with multinational findings regarding CBCL-DP trajectories associated with individual psychosocial adversities.^11,17,29^

Previous findings have varied depending on substance exposures, risk factors, and covariates included in multivariable models. Earlier work using confirmed frequency, timing, duration, and/or toxicological exposure measures reported greater CBCL problems associated with opioid,^14^ cocaine,^16^ marijuana,^63^ nicotine,^64^ alcohol,^65,66^ and polysubstance exposures.^67,68^ The finding that alcohol exposure was unexpectedly highest in the low dysregulation class warrants further study, where frequency, timing, and duration can be examined precisely for all exposures used.^6^

Importantly, high and borderline dysregulation trajectories were more prevalent among children with combined increases in both prenatal substance exposures and psychosocial adversities. Mothers with any psychological history had increased substance use and psychosocial adversities; prior diagnoses were most prevalent among those who used opioids or illicit substances (eTables 3 and 4 in Supplement 1). This combination of substance use and psychosocial adversities represents a subset of pregnant individuals who may benefit from high-quality, accessible diagnostic and targeted intervention services, ideally beginning with preconception care.^69,70^

In summary, our findings address gaps by specifying early risk factors and the antecedent behavioral problems associated with long-term dysregulation. Risk and protective factors identified here can be ascertained prenatally, at birth, and prior to postnatal discharge to the home community to individualize family supports.^71,72^ When initiated earlier, behavioral and emotional interventions are more effective,^73,74,75,76,77^ with potential to prevent escalation among children with borderline symptoms. Toward the goal of disrupting persisting dysregulation and facilitating resilience, the cumulative risks and behavioral indicators identified inform best practices and future research.

### Strengths and Limitations

Study strengths include the large, diverse multicohort sample, with rigorously selected measures and data collection procedures implemented ECHO-wide to examine multiple characteristics of children, families, and environments. Importantly, 92.8% of cohort parents consented to ECHO; 7.2% refused, aged out, or were unreachable. Similarly impressive is the low 1.3% active withdrawal rate.

While maternal and environmental characteristics across the study period would be useful to study in future work, our focus was on the earliest and most salient risk factors to best inform preventive and targeted screening and intervention practices. We therefore used repeated CBCL measures to strengthen screening specificity by documenting antecedents of persisting dysregulation trajectories reflected in CBCL subscores.^60,61^ Furthermore, our findings include early risk factors for persisting dysregulation among young US children with highly diverse family and health backgrounds.

This study also had limitations. Despite evidence of reliable self-reporting and convergence with bioassays,^36,37,38^ limitations are the likely underreporting of substance exposures and the absence of quantified frequencies, durations, and amounts. Missing covariates due to cohort data collection variations required mitigation by imputation, and LOS among preterms was used as an informative proxy for neonatal illness severity indicated by extended stays.

## Conclusions

This cohort study identified antecedents associated with resilience and risk in dysregulation pathways across the first 6 years of life. These findings warrant support for children with early indicated needs for monitoring and targeted interventions to address risk for persisting dysregulation.^78,79^

## Notes

Supplement 1. **eFigure 1.** Prenatal Single Substance and Polysubstance Exposure Patterns Among ECHO Children **eFigure 2.** Distribution of ECHO Cohorts’ Participant Recruitment Sites Across the United States **eTable 1.** Cohort Characteristics **eTable 2.** Growth Mixture Modeling Fit Statistics **eTable 3.** Prenatal Substance Use Among Mothers With Prior Psychiatric Diagnosis and Positive PROMIS Depression Screen **eTable 4.** Distribution of Psychosocial Adversity Index Variables Among Mothers With Versus Without a Prior Psychiatric Diagnosis and/or Positive Depressive Symptom Screen

Supplement 2.
Nonauthor Collaborators

Supplement 3.
Data Sharing Statement

## References

[1] Rescorla LA, Jordan P, Zhang S, Baelen-King G, Althoff RR, Ivanova MY; International Aseba Consortium. Latent class analysis of the CBCL dysregulation profile for 6- to 16-year-olds in 29 societies. J Clin Child Adolesc Psychol. 2021;50(5):551-564. doi:10.1080/15374416.2019.1697929 31914322

[2] Althoff RR, Verhulst FC, Rettew DC, Hudziak JJ, van der Ende J. Adult outcomes of childhood dysregulation: a 14-year follow-up study. J Am Acad Child Adolesc Psychiatry. 2010;49(11):1105-1116. doi:10.1097/00004583-201011000-00004 20970698 PMC2965164

[3] Biederman J, Petty CR, Monuteaux MC, . The Child Behavior Checklist-Pediatric Bipolar Disorder profile predicts a subsequent diagnosis of bipolar disorder and associated impairments in ADHD youth growing up: a longitudinal analysis. J Clin Psychiatry. 2009;70(5):732-740. doi:10.4088/JCP.08m04821 19389330 PMC3066229

[4] Meyer SE, Carlson GA, Youngstrom E, . Long-term outcomes of youth who manifested the CBCL-Pediatric Bipolar Disorder phenotype during childhood and/or adolescence. J Affect Disord. 2009;113(3):227-235. doi:10.1016/j.jad.2008.05.024 18632161

[5] Kim DR, Bale TL, Epperson CN. Prenatal programming of mental illness: current understanding of relationship and mechanisms. Curr Psychiatry Rep. 2015;17(2):5-5. doi:10.1007/s11920-014-0546-9 25617041 PMC4458064

[6] Dukes K, Tripp T, Willinger M, ; PASS Network. Drinking and smoking patterns during pregnancy: Development of group-based trajectories in the Safe Passage Study. Alcohol. 2017;62:49-60. doi:10.1016/j.alcohol.2017.03.001 28755751 PMC5604849

[7] Barrios YV, Maselko J, Engel SM, . The relationship of cumulative psychosocial adversity with antepartum depression and anxiety. Depress Anxiety. 2021;38(10):1034-1045. doi:10.1002/da.23206 34370895

[8] Evans GW, Kim P. Childhood poverty and young adults’ allostatic load: the mediating role of childhood cumulative risk exposure. Psychol Sci. 2012;23(9):979-983. doi:10.1177/0956797612441218 22825357

[9] Klass P. Saving Tiny Tim–Pediatrics and Childhood Poverty in the United States. N Engl J Med. 2016;374(23):2201-2205. doi:10.1056/NEJMp1603516 27276559

[10] Racine N, McArthur BA, Cooke JE, Eirich R, Zhu J, Madigan S. Global prevalence of depressive and anxiety symptoms in children and adolescents during COVID-19: a meta-analysis. JAMA Pediatr. 2021;175(11):1142-1150. doi:10.1001/jamapediatrics.2021.2482 34369987 PMC8353576

[11] Asmussen J, Skovgaard AM, Bilenberg N. Trajectories of dysregulation in preschool age. Eur Child Adolesc Psychiatry. 2022;31(2):313-324. doi:10.1007/s00787-020-01689-z 33386524

[12] O’Donnell KJ, Glover V, Barker ED, O’Connor TG. The persisting effect of maternal mood in pregnancy on childhood psychopathology. Dev Psychopathol. 2014;26(2):393-403. doi:10.1017/S0954579414000029 24621564

[13] Solis JM, Shadur JM, Burns AR, Hussong AM. Understanding the diverse needs of children whose parents abuse substances. Curr Drug Abuse Rev. 2012;5(2):135-147. doi:10.2174/1874473711205020135 22455509 PMC3676900

[14] Bauer CR, Langer J, Lambert-Brown B, . Association of prenatal opiate exposure with youth outcomes assessed from infancy through adolescence. J Perinatol. 2020;40(7):1056-1065. doi:10.1038/s41372-020-0692-3 32444681

[15] Chu EK, Smith LM, Derauf C, . Behavior problems during early childhood in children with prenatal methamphetamine exposure. Pediatrics. 2020;146(6):e20190270. doi:10.1542/peds.2019-0270 33172920 PMC7706113

[16] Min MO, Albert JM, Lorincz-Comi N, . Prenatal substance exposure and developmental trajectories of internalizing symptoms: toddlerhood to preadolescence. Drug Alcohol Depend. 2021;218:108411. doi:10.1016/j.drugalcdep.2020.108411 33272717 PMC7750298

[17] Lean RE, Lessov-Shlaggar CN, Gerstein ED, . Maternal and family factors differentiate profiles of psychiatric impairments in very preterm children at age 5-years. J Child Psychol Psychiatry. 2020;61(2):157-166. doi:10.1111/jcpp.13116 31449335 PMC6980170

[18] Johnson S, Marlow N. Growing up after extremely preterm birth: lifespan mental health outcomes. Semin Fetal Neonatal Med. 2014;19(2):97-104. doi:10.1016/j.siny.2013.11.004 24290907

[19] Anderson PJ, de Miranda DM, Albuquerque MR, . Psychiatric disorders in individuals born very preterm / very low-birth weight: an individual participant data (IPD) meta-analysis. EClinicalMedicine. 2021;42:101216-101216. doi:10.1016/j.eclinm.2021.101216 34901794 PMC8639417

[20] Linsell L, Johnson S, Wolke D, Morris J, Kurinczuk JJ, Marlow N. Trajectories of behavior, attention, social and emotional problems from childhood to early adulthood following extremely preterm birth: a prospective cohort study. Eur Child Adolesc Psychiatry. 2019;28(4):531-542. doi:10.1007/s00787-018-1219-8 30191335 PMC6445809

[21] Arpi E, Ferrari F. Preterm birth and behaviour problems in infants and preschool-age children: a review of the recent literature. Dev Med Child Neurol. 2013;55(9):788-796. doi:10.1111/dmcn.12142 23521214

[22] Johnson S, Waheed G, Manktelow BN, . Differentiating the preterm phenotype: distinct profiles of cognitive and behavioral development following late and moderately preterm birth. J Pediatr. 2018;193:85-92.e1. doi:10.1016/j.jpeds.2017.10.002 29254758

[23] Evans GW, Li D, Whipple SS. Cumulative risk and child development. Psychol Bull. 2013;139(6):1342-1396. doi:10.1037/a0031808 23566018

[24] Kim J, Carlson GA, Meyer SE, . Correlates of the CBCL-dysregulation profile in preschool-aged children. J Child Psychol Psychiatry. 2012;53(9):918-926. doi:10.1111/j.1469-7610.2012.02546.x 22409304 PMC3523168

[25] Achenbach TM, Rescorla LA. Manual for the ASEBA preschool forms and profiles. Vol 30. University of Vermont, Research center for children, youth; 2000.

[26] Basten M, Tiemeier H, Althoff RR, . The stability of problem behavior across the preschool years: an empirical approach in the general population. J Abnorm Child Psychol. 2016;44(2):393-404. doi:10.1007/s10802-015-9993-y 25832625 PMC4729812

[27] Althoff RR, Ayer LA, Crehan ET, Rettew DC, Baer JR, Hudziak JJ. Temperamental profiles of dysregulated children. Child Psychiatry Hum Dev. 2012;43(4):511-522. doi:10.1007/s10578-012-0280-7 22271225 PMC3374895

[28] Reef J, van Meurs I, Verhulst FC, van der Ende J. Children’s problems predict adults’ DSM-IV disorders across 24 years. J Am Acad Child Adolesc Psychiatry. 2010;49(11):1117-1124. doi:10.1016/j.jaac.2010.08.002 20970699

[29] de Lijster JM, van den Dries MA, van der Ende J, . Developmental trajectories of anxiety and depression symptoms from early to middle childhood: a population-based cohort study in the Netherlands. J Abnorm Child Psychol. 2019;47(11):1785-1798. doi:10.1007/s10802-019-00550-5 31069583 PMC6805800

[30] Althoff RR, Rettew DC, Faraone SV, Boomsma DI, Hudziak JJ. Latent class analysis shows strong heritability of the child behavior checklist-juvenile bipolar phenotype. Biol Psychiatry. 2006;60(9):903-911. doi:10.1016/j.biopsych.2006.02.025 16650832

[31] Holtmann M, Buchmann AF, Esser G, Schmidt MH, Banaschewski T, Laucht M. The Child Behavior Checklist-Dysregulation Profile predicts substance use, suicidality, and functional impairment: a longitudinal analysis. J Child Psychol Psychiatry. 2011;52(2):139-147. doi:10.1111/j.1469-7610.2010.02309.x 20854363

[32] Denckla CA, Cicchetti D, Kubzansky LD, . Psychological resilience: an update on definitions, a critical appraisal, and research recommendations. Eur J Psychotraumatol. 2020;11(1):1822064. doi:10.1080/20008198.2020.1822064 33244362 PMC7678676

[33] Gillman MW, Blaisdell CJ. Environmental influences on Child Health Outcomes, a Research Program of the National Institutes of Health. Curr Opin Pediatr. 2018;30(2):260-262. doi:10.1097/MOP.0000000000000600 29356702 PMC6020137

[34] Knapp EA, Kress AM, Parker CB, . The Environmental influences on Child Health Outcomes (ECHO)-wide Cohort. Am J Epidemiol. Published online March 24, 2023. doi:10.1093/aje/kwad071 PMC10403303 36963379

[35] Romano ME, Buckley JP, Elliott AJ, Johnson CC, Paneth N. SPR Perspectives: scientific opportunities in the Environmental influences on Child Health Outcomes Program. Pediatr Res. 2022;92(5):1255-1261. 34035428 10.1038/s41390-021-01577-5 PMC8145190

[36] Neiderhiser JM, Marceau K, De Araujo-Greecher M, . Estimating the roles of genetic risk, perinatal risk, and marital hostility on early childhood adjustment: medical records and self-reports. Behav Genet. 2016;46(3):334-352. doi:10.1007/s10519-016-9788-0 27075497 PMC4860070

[37] Ramos AM, Marceau K, Neiderhiser JM, De Araujo-Greecher M, Natsuaki MN, Leve LD. Maternal consistency in recalling prenatal experiences at 6 months and 8 years postnatal. J Dev Behav Pediatr. 2020;41(9):698-705. doi:10.1097/DBP.0000000000000841 32740284 PMC7942020

[38] Pickett KE, Kasza K, Biesecker G, Wright RJ, Wakschlag LS. Women who remember, women who do not: a methodological study of maternal recall of smoking in pregnancy. Nicotine Tob Res. 2009;11(10):1166-1174. doi:10.1093/ntr/ntp117 19640836 PMC2746835

[39] Humeniuk R, Ali R, Babor TF, . Validation of the Alcohol, Smoking And Substance Involvement Screening Test (ASSIST). Addiction. 2008;103(6):1039-1047. doi:10.1111/j.1360-0443.2007.02114.x 18373724

[40] Carta JJ, Atwater JB, Greenwood CR, McConnell SR, McEvoy MA, Williams R. Effects of cumulative prenatal substance exposure and environmental risks on children’s developmental trajectories. J Clin Child Psychol. 2001;30(3):327-337. doi:10.1207/S15374424JCCP3003_5 11501250

[41] Kroenke K, Stump TE, Chen CX, . Minimally important differences and severity thresholds are estimated for the PROMIS depression scales from three randomized clinical trials. J Affect Disord. 2020;266:100-108. doi:10.1016/j.jad.2020.01.101 32056864 PMC7103541

[42] Choi SW, Podrabsky T, McKinney N, Schalet BD, Cook KF. PROsetta Stone® Methodology: a Rosetta Stone for patient reported outcomes. Department of Medical Social Sciences, Feinberg School of Medicine, Northwestern University. Published 2012. Accessed March 21, 2023. https://static1.squarespace.com/static/60c7c36a1afd4a3ab90af0a6/t/60c8f437fa0dd42417acbd6e/1623782456126/PROSetta+Methodology+Report.pdf

[43] Choi SW, Lim S, Schalet BD, Kaat AJ, Cella D. PROsetta: an *R* package for linking patient-reported outcome measures. Appl Psychol Meas. 2021;45(5):386-388. doi:10.1177/01466216211013106 34565942 PMC8361372

[44] Blackwell CK, Tang X, Elliott AJ, . Developing a common metric for depression across adulthood: Linking PROMIS depression with the Edinburgh Postnatal Depression Scale. Psychol Assess. 2021;33(7):610-618. doi:10.1037/pas0001009 34060864 PMC8284177

[45] Choi SW, Schalet B, Cook KF, Cella D. Establishing a common metric for depressive symptoms: linking the BDI-II, CES-D, and PHQ-9 to PROMIS depression. Psychol Assess. 2014;26(2):513-527. doi:10.1037/a0035768 24548149 PMC5515387

[46] Sunderland M, Batterham P, Calear A, Carragher N. Validity of the PROMIS depression and anxiety common metrics in an online sample of Australian adults. Qual Life Res. 2018;27(9):2453-2458. doi:10.1007/s11136-018-1905-5 29872956

[47] Cella D, Schalet BD, Kallen M, . PROMIS Depression and Kessler Psychological Distress Scale (K6) 2016. Accessed August 1, 2022. https://static1.squarespace.com/static/60c7c36a1afd4a3ab90af0a6/t/60d8a1ae14fd3e66d3232e84/1624809903375/PROMIS+Depression+and+K6+Full+Report.pdf

[48] Roubinov D, Musci RJ, Hipwell AE, . Trajectories of depressive symptoms among mothers of preterm and full-term infants in a national sample. Arch Womens Ment Health. 2022;25(4):807-817. doi:10.1007/s00737-022-01245-5 35708790 PMC9283322

[49] Sameroff AJ, Seifer R, Barocas R, Zax M, Greenspan S. Intelligence quotient scores of 4-year-old children: social-environmental risk factors. Pediatrics. 1987;79(3):343-350. doi:10.1542/peds.79.3.343 3822634

[50] Shrider EA, Kollar M, Chen F, Semega J. U.S. Census Bureau. Income and poverty in the United States: 2020. current population reports. US Census Bureau. Published September 2021. Accessed March 29, 2023. https://www.census.gov/content/dam/Census/library/publications/2021/demo/p60-273.pdf

[51] Papageorghiou AT, Kennedy SH, Salomon LJ, ; International Fetal and Newborn Growth Consortium for the 21(st) Century (INTERGROWTH-21(st)). The INTERGROWTH-21st fetal growth standards: toward the global integration of pregnancy and pediatric care. Am J Obstet Gynecol. 2018;218(2S):S630-S640. doi:10.1016/j.ajog.2018.01.011 29422205

[52] Frazier JA, Wood ME, Ware J, ; ELGAN Study Investigators. Antecedents of the child behavior checklist-dysregulation profile in children born extremely preterm. J Am Acad Child Adolesc Psychiatry. 2015;54(10):816-823. doi:10.1016/j.jaac.2015.07.008 26407491 PMC4615708

[53] Nylund-Gibson K, Grimm R, Quirk M, Furlong M. A Latent Transition Mixture Model Using the Three-Step Specification. Struct Equ Modeling. 2014;21(3):439-454. doi:10.1080/10705511.2014.915375

[54] Wang M, Bodner TE. Growth mixture modeling: identifying and predicting unobserved subpopulations with longitudinal data. Organ Res Methods. 2007;10(4):635-656. doi:10.1177/1094428106289397

[55] Masyn KE. Latent class analysis and finite mixture modeling. In: Little TD, ed. The Oxford Handbook of Quantitative Methods: Statistical Analysis. Oxford University Press; 2013:551-611.

[56] Little TD, Jorgensen TD, Lang KM, Moore EW. On the joys of missing data. J Pediatr Psychol. 2014;39(2):151-162. doi:10.1093/jpepsy/jst048 23836191

[57] Graham JW. Adding missing-data-relevant variables to FIML-based structural equation models. Struct Equ Modeling. 2003;10(1):80-100. doi:10.1207/S15328007SEM1001_4

[58] Enders CK, Bandalos DL. The relative performance of full information maximum likelihood estimation for missing data in structural equation models. Struct Equ Modeling. 2001;8(3):430-457. doi:10.1207/S15328007SEM0803_5

[59] Muthen L, Muthen B. Mplus user’s guide 8th edition 1998-2017*.* Muthen & Muthen. Accessed March 21, 2023. https://www.statmodel.com/download/usersguide/MplusUserGuideVer_8.pdf

[60] Dölitzsch C, Kölch M, Fegert JM, Schmeck K, Schmid M. Ability of the Child Behavior Checklist-Dysregulation Profile and the Youth Self Report-Dysregulation Profile to identify serious psychopathology and association with correlated problems in high-risk children and adolescents. J Affect Disord. 2016;205:327-334. doi:10.1016/j.jad.2016.08.010 27566452

[61] Basten MM, Althoff RR, Tiemeier H, . The dysregulation profile in young children: empirically defined classes in the Generation R study. J Am Acad Child Adolesc Psychiatry. 2013;52(8):841-850.e2. doi:10.1016/j.jaac.2013.05.007 23880494 PMC3813902

[62] Bogičević L, Verhoeven M, van Baar AL. Toddler skills predict moderate-to-late preterm born children’s cognition and behaviour at 6 years of age. PLoS One. 2019;14(11):e0223690. doi:10.1371/journal.pone.0223690 31693682 PMC6834277

[63] Goldschmidt L, Day NL, Richardson GA. Effects of prenatal marijuana exposure on child behavior problems at age 10. Neurotoxicol Teratol. 2000;22(3):325-336. doi:10.1016/S0892-0362(00)00066-0 10840176

[64] Eiden RD, Zhao J, Casey M, Shisler S, Schuetze P, Colder CR. Pre- and postnatal tobacco and cannabis exposure and child behavior problems: bidirectional associations, joint effects, and sex differences. Drug Alcohol Depend. 2018;185:82-92. doi:10.1016/j.drugalcdep.2017.11.038 29428324 PMC5889743

[65] Long X, Lebel C. Evaluation of Brain Alterations and Behavior in Children With Low Levels of Prenatal Alcohol Exposure. JAMA Netw Open. 2022;5(4):e225972. doi:10.1001/jamanetworkopen.2022.5972 35380644 PMC8984786

[66] Behnke M, Smith VC; Committee on Substance Abuse; Committee on Fetus and Newborn. Prenatal substance abuse: short- and long-term effects on the exposed fetus. Pediatrics. 2013;131(3):e1009-e1024. doi:10.1542/peds.2012-3931 23439891 PMC8194464

[67] Min MO, Minnes S, Park H, . Developmental trajectories of externalizing behavior from ages 4 to 12: prenatal cocaine exposure and adolescent correlates. Drug Alcohol Depend. 2018;192:223-232. doi:10.1016/j.drugalcdep.2018.08.007 30273890 PMC6310164

[68] Bada HS, Das A, Bauer CR, . Impact of prenatal cocaine exposure on child behavior problems through school age. Pediatrics. 2007;119(2):e348-e359. doi:10.1542/peds.2006-1404 17272597

[69] Jones HE, Apsley HB, Cocowitch A, . Increasing knowledge about recovery-related life domains among pregnant and parenting women in comprehensive substance use disorder treatment: The Art of Addiction Recovery Program. Drug Alcohol Depend. 2022;232:109252. doi:10.1016/j.drugalcdep.2021.109252 35032855 PMC8885977

[70] Klaman SL, Isaacs K, Leopold A, . Treating women who are pregnant and parenting for opioid use disorder and the concurrent care of their infants and children: literature review to support national guidance. J Addict Med. 2017;11(3):178-190. doi:10.1097/ADM.0000000000000308 28406856 PMC5457836

[71] Milgrom J, Hirshler Y, Reece J, Holt C, Gemmill AW. Social support-a protective factor for depressed perinatal women? Int J Environ Res Public Health. 2019;16(8):1426. doi:10.3390/ijerph16081426 31010090 PMC6518117

[72] Kaminski JW, Robinson LR, Hutchins HJ, Newsome KB, Barry CM. Evidence base review of couple- and family-based psychosocial interventions to promote infant and early childhood mental health, 2010-2019. J Marital Fam Ther. 2022;48(1):23-55. doi:10.1111/jmft.12570 34783041 PMC10995740

[73] Bagner DM, Berkovits MD, Coxe S, . Telehealth treatment of behavior problems in young children with developmental delay: a randomized clinical trial. JAMA Pediatr. 2023;177(3):231-239. doi:10.1001/jamapediatrics.2022.5204 36622653 PMC9857733

[74] Bastain TM, Knapp EA, Law A, ; Environmental Influences on Child Health Outcomes Program Collaborators. COVID-19 pandemic experiences and symptoms of pandemic-associated traumatic stress among mothers in the US. JAMA Netw Open. 2022;5(12):e2247330. doi:10.1001/jamanetworkopen.2022.47330 36525271 PMC9856510

[75] Welch MG, Firestein MR, Austin J, . Family nurture intervention in the neonatal intensive care unit improves social-relatedness, attention, and neurodevelopment of preterm infants at 18 months in a randomized controlled trial. J Child Psychol Psychiatry. 2015;56(11):1202-1211. doi:10.1111/jcpp.12405 25763525

[76] McKinney J, Keyser L, Clinton S, Pagliano C. ACOG Committee Opinion No. 736: optimizing postpartum care. Obstet Gynecol. 2018;132(3):784-785. doi:10.1097/AOG.0000000000002849 30134408

[77] Milgrom J, Schembri C, Ericksen J, Ross J, Gemmill AW. Towards parenthood: an antenatal intervention to reduce depression, anxiety and parenting difficulties. J Affect Disord. 2011;130(3):385-394. doi:10.1016/j.jad.2010.10.045 21112641

[78] Guttentag CL, Landry SH, Williams JM, . “My Baby & Me”: effects of an early, comprehensive parenting intervention on at-risk mothers and their children. Dev Psychol. 2014;50(5):1482-1496. doi:10.1037/a0035682 24447116 PMC5609813

[79] O’Farrelly C, Watt H, Babalis D, . A brief home-based parenting intervention to reduce behavior problems in young children: a pragmatic randomized clinical trial. JAMA Pediatr. 2021;175(6):567-576. doi:10.1001/jamapediatrics.2020.6834 33720329 PMC7961467

