# Supplementary material for: Assessment of Psychosocial and Neonatal Risk Factors for Trajectories of Behavioral Dysregulation Among Young Children From 18 to 72 Months of Age
Source: JAMA Netw Open. 2023 Apr 26;6(4):e2310059. doi: 10.1001/jamanetworkopen.2023.10059 (PMC10134008; doi:10.1001/jamanetworkopen.2023.10059)
Supplement: Supplement 1. — eFigure 1. Prenatal Single Substance and Polysubstance Exposure Patterns Among ECHO Children eFigure 2. Distribution of ECHO Cohorts’ Participant Recruitment Sites Across the United States eTable 1. Cohort Characteristics eTable 2. Growth Mixture Modeling Fit Statistics eTable 3. Prenatal Substance Use Among Mothers With Prior Psychiatric Diagnosis and Positive PROMIS Depression Screen eTable 4. Distribution of Psychosocial Adversity Index Variables Among Mothers With Versus Without a Prior Psychiatric Diagnosis and/or Positive Depressive Symptom Screen [file jamanetwopen-e2310059-s001.pdf]

Supplemental Online Content

Hofheimer JA, McGrath M, Musci R, et al; Program Collaborators for Environmental influences on Child Health Outcomes. Assessment of psychosocial and neonatal risk factors for trajectories of behavioral dysregulation among young children from 18 to 72 months of age. *JAMA Netw Open.* 2023;6(4):e2310059. doi:10.1001/jamanetworkopen.2023.10059

eFigure 1. Prenatal Single Substance and Polysubstance Exposure Patterns Among ECHO Children

eFigure 2. Distribution of ECHO Cohorts' Participant Recruitment Sites Across the United States

eTable 1. Cohort Characteristics

eTable 2. Growth Mixture Modeling Fit Statistics

eTable 3. Prenatal Substance Use Among Mothers With Prior Psychiatric Diagnosis and Positive PROMIS Depression Screen

eTable 4. Distribution of Psychosocial Adversity Index Variables Among Mothers With Versus Without a Prior Psychiatric Diagnosis and/or Positive Depressive Symptom Screen

This supplemental material has been provided by the authors to give readers additional information about their work.

© 2023 Hofheimer JA et al. *JAMA Network Open.*

eFigure 1. Prenatal Single Substance[a] and Polysubstance Exposure Patterns Among ECHO Children

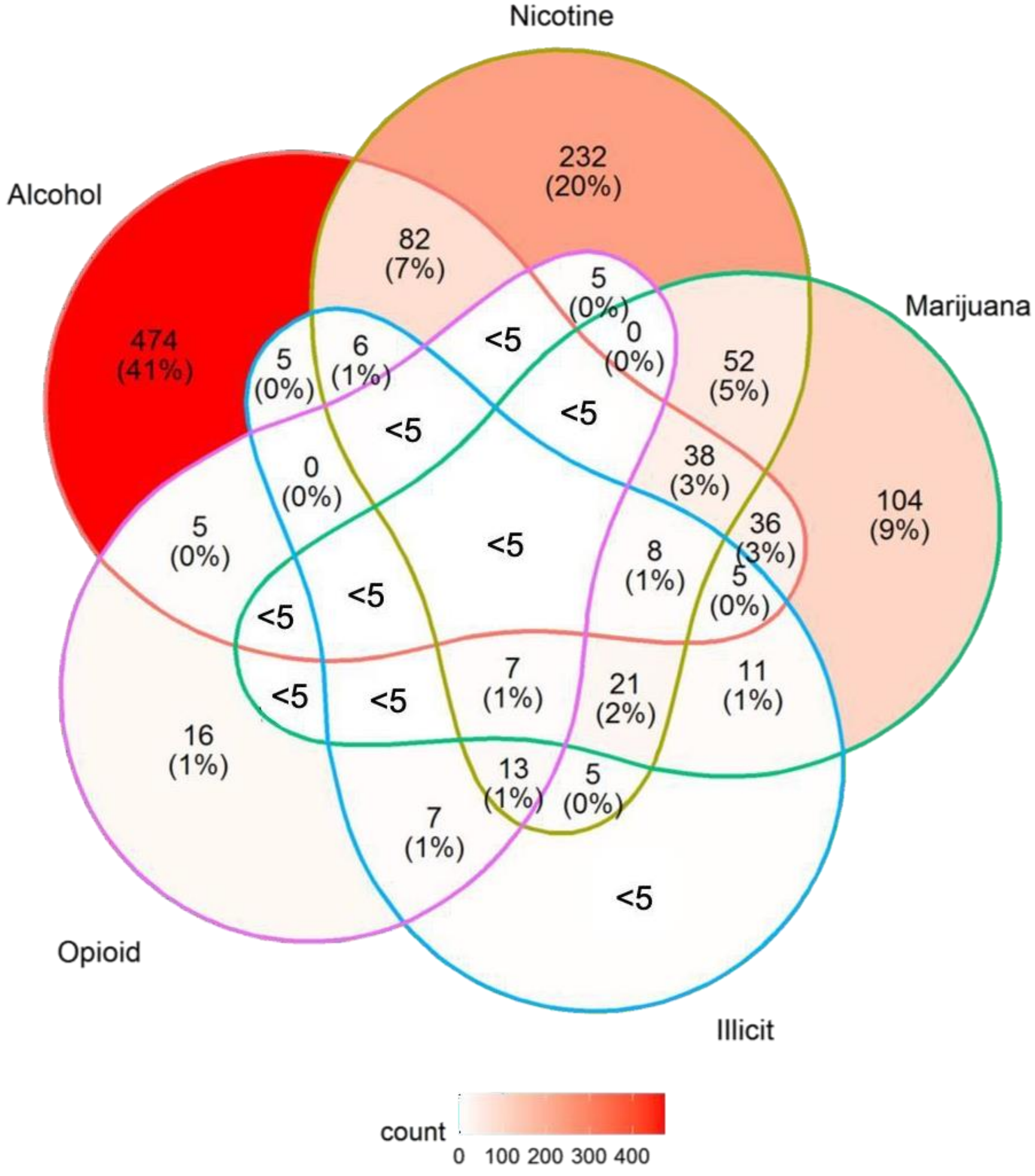

[a]Illicit substances: cocaine, heroin, methamphetamines, hallucinogens, inhalants, or confirmed use of unprescribed or misused pharmaceutical (e.g., amphetamines, benzodiazepines, ketamine).

© 2023 Hofheimer JA et al. *JAMA Network Open.*

eFigure 2. Distribution of ECHO Cohorts' Participant Recruitment Sites Across the United States

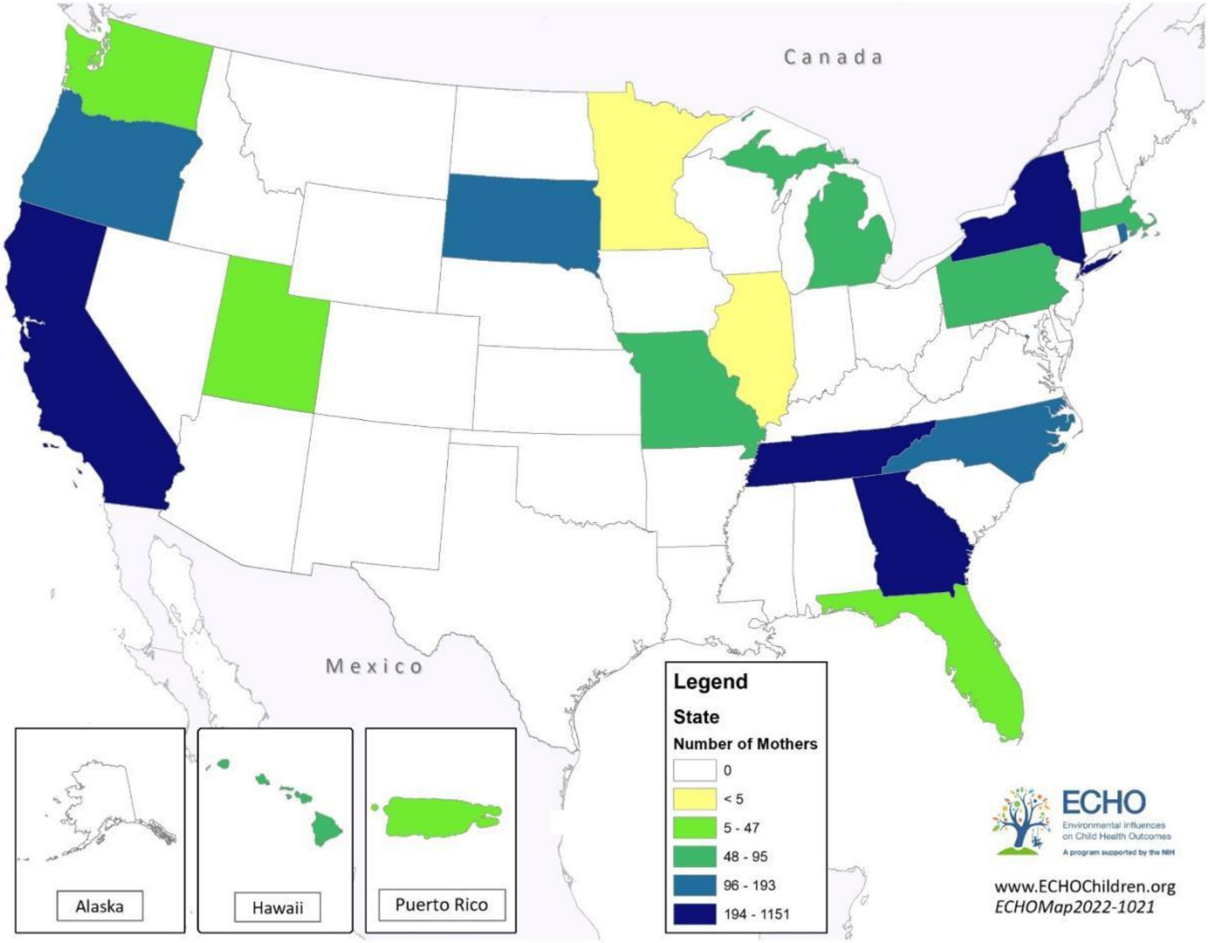

© 2023 Hofheimer JA et al. *JAMA Network Open.*

eTable 1. Cohort Characteristics[1]

Cohort	Sampling, inclusion, and exclusion criteria	Study aims and assessments
A	From 2011-2013 and in 2015, very low birth weight (<1500 g) infants were enrolled. Inclusion criteria: 1. birthweight under 1500 g or gestational age 28-0/7 through 32-6/7 weeks, 2. birth at Mount Sinai Hospital during the time period of study enrollment (2011-13; March 2015-present), 3. no diagnosis of genetic or major structural congenital abnormality (heart, lung, kidney, intestinal). Exclusion criteria: evidence of perinatal hypoxic ischemic encephalopathy.	The aim is to identify and assess the impact of NICU-based chemical and non-chemical environmental exposures on neurobehavioral outcomes through early childhood. Exposures measured in urine, meconium, stool, hair, saliva, and blood spots hypothesized to be associated with neurodevelopmental outcomes on the NICU Network Neurobehavioral Scale, Child Behavior Checklist (CBCL), and Bayley scales.
B	This cohort comprises participants from a multicenter, randomized placebo-controlled phase III 940-subject trial of erythropoietin for the neuroprotection of extremely low gestational age neonates (born at <28 weeks). Inclusion criteria: 1. NICU inpatients between 24-0/7 and 27-6/7 weeks of gestation, 2. 24 hours of age or less, 3. arterial or venous access, 4. parental consent. Exclusion criteria: 1. known major life-threatening anomalies (e.g., fetal diagnosis of brain, cardiac, or renal malformation); 2. known or suspected chromosomal anomalies; 3. severe hematologic crises, such as disseminated intravascular coagulopathy, twin-twin transfusion; 4. polycythemia (hematocrit >65%); 5. hydrops fetalis; 6. congenital infection, such as toxoplasmosis, cytomegalovirus (CMV), rubella, or syphilis; 7. prior administration of erythropoietin to the baby.	The aim of PENUT was to examine erythropoietin effects on trial outcomes that included the CBCL.
C	General population of children recruited between 3 months and 12 years of age. Inclusion criteria: 1. healthy children, 2. no metal in body (safe for magnetic resonance imaging [MRI] component of study), 3. no diagnosis of attention-deficit/hyperactivity disorder (ADHD); 4. no older than 12 years of age. Exclusion criteria: 1. children with major risk factors for brain abnormalities; 2. in utero alcohol, cigarette, or illicit substance exposure; 3. preterm (<37 weeks gestation) birth; 4. small for gestational age or less than 1500 g; 5. fetal ultrasound abnormalities; 6. complicated pregnancy, including preeclampsia, high blood pressure, and gestational diabetes mellitus (GDM); 7. APGAR scores <8; 8. NICU admission; 9. neurological disorder (e.g., head injury, epilepsy); 10. psychiatric or learning disorder in the infant, parents, or siblings (such as medicated depression).	Longitudinal MRI and cognitive data were collected among children 3 months to 12 years of age to examine neuropsychological and behavioral development, including CBCLs, myelin maturation patterns, and APOE genetic information.

[1]*The ECHO-WIDE Data Collection Protocol includes assessment procedures for exposures and outcomes : https://dcricollab.dcri.duke.edu/sites/echomaterials/MOP%20and%20Protocol/ECHOwide%20Cohort%20Data%20Collection%20Protocol%20Version%202.11.pdf*

© 2023 Hofheimer JA et al. *JAMA Network Open.*

Cohort	Sampling, inclusion, and exclusion criteria	Study aims and assessments
D	General population of children recruited between 3 months and 12 years of age. Inclusion criteria: 1. pregnant mothers with uncomplicated pregnancies; 2. no alcohol, cigarette, or illicit substance use (including marijuana); 3. singleton pregnancy; 4. no abnormalities on first trimester fetal ultrasound; 5. no major psychiatric disorder (including depression requiring medication); 6. preterm (<37 weeks) birth; 7. no NICU admission; 8. no history of neurological disorder (e.g., head injury, epilepsy) in infant; 9. no history of psychiatric or learning disorder in the infant, parents, or siblings. Exclusion criteria: none listed.	This study examined pre- and post-natal relationships between metabolic changes, physical growth, and brain development with CBCL and other protocol assessments.
E	Enrollment in 2001-2002 of 2,491 Latino youth (age 5-13 years). Participants were living in the South Bronx, New York City and San Juan, Puerto Rico. Inclusion criteria: participation in the Boricua Youth Study Wave 1 (BYS W1), which required: 1. being 5-13 years of age in 2001-2002, 2. living in South Bronx, New York or San Juan Metropolitan Area and Caguas, Puerto Rico in 2001-2002, 3. being of Puerto Rican background at enumeration of BYS W1, 4. having at least one caretaker identify as Puerto Rican. Exclusion criteria: 1. youth known to be severely impaired neuropsychologically, 2. youth who are permanently institutionalized or youth who have run away or left home, 3. emancipated youth whose parent cannot provide consent.	This epidemiological study of antisocial behaviors examines key factors that influence intergenerational socioeconomic disadvantage and the impact of adversity on child neurodevelopmental and behavioral (CBCL) health and functioning.
F	This prenatal cohort of pregnant African American individuals in Atlanta, Georgia without chronic health conditions enrolled participants between 8-14 weeks' gestation in 2014. Inclusion criteria: 1. African American (self-identified as Black/African American and self-report as United States-born), 2. 18-40 years of age at the time of enrollment, 3. singleton pregnancy between 8-14 weeks; 4. fewer than five previous births, 5. no chronic medical conditions and not taking prescription medications chronically. Exclusion criteria: 1. post-enrollment: intrauterine death; 2. post-enrollment: congenital anomalies.	Aims are to examine the role of the microbiome in preterm birth. CBCL and other protocol assessments were completed to identify intrauterine and early childhood environmental exposures and risk pathways contributing to children’s neurodevelopmental deficits and obesity.
G	This cohort includes both an extant cohort from the Prenatal Alcohol in SIDS and Stillbirth Research Network (PASS [N=12,029]) and a new pregnancy cohort of White and American Indian women recruited in pregnancy from participating obstetrics and gynecology clinics and other entities (N=1,699). Inclusion criteria: 1. pregnant female of any race or ethnicity carrying one or two fetuses during pregnancy; 2. age 16 years or older, inclusive at time of consent; 3. at time of recruitment visit, participant is at least 6 weeks, 0 days and <20 weeks, 1 day gestation - OR participant is 20 weeks, 1 day gestation, has not had more than two prenatal visits - AND the current visit is not the delivery admission; 6. must speak English (Northern Plains only); 7. able to provide informed consent. Exclusion criteria: 1. women carrying three or more fetuses during the pregnancy, 2. planned abortion, 3. moving out of catchment area prior to estimated date of delivery, 4. unable to provide informed consent, 5. health care provider advises against participation.	Aims are to examine the relationship between prenatal alcohol exposure associated with asthma and behavioral (CBCL), neurodevelopmental, and neurophysiological outcomes.

© 2023 Hofheimer JA et al. *JAMA Network Open.*

Cohort	Sampling, inclusion, and exclusion criteria	Study aims and assessments
H	PETALS recruited a prospective cohort of 3,350 pregnant individuals from four California hospitals. Inclusion criteria: 1. pregnant women; 2. current members at Kaiser Permanente Northern California. Exclusion criteria: 1. age <18 years, 2. pre-existing diabetes diagnosis, 3. severe liver disease (hepatitis C, cirrhosis), 4. multiple pregnancy.	Study aims are to examine whether BPA levels are associated with GDM and excessive infant birthweight, growth, and development. Follow-up outcome assessments included the CBCL.
I	The Kaiser Permanente Research Bank (KPRB) Pregnancy Cohort enrolled pregnant individuals and integrated these assessments into routine prenatal medical care. Inclusion criteria: 1. current Kaiser Permanente Northern California (KPNC) female member, 2. >= 18 years of age, 3. currently receiving prenatal care at KPNC facility. Exclusion criteria: 1. <18 years of age; 2. not a KPNC member currently, 3. male, 4. not pregnant.	The KPNC developed a genetic epidemiology population resource to examine associations between environmental exposures and follow-up assessments of infant outcomes including the CBCL.
J	CANDLE enrolled 1,503 pregnant individuals who delivered between 2006- 2011 at four hospitals in Memphis, Tennessee (TN); 1,385 mother-child dyads are still under active follow-up. The cohort is 63% African American, and 64% of the cohort have an annual income <$50,000. Inclusion criteria: 1. Shelby County, TN resident, 2. pregnancy 16-28 weeks gestation (at enrollment), 3. ages 16-40 years; 4. speak and understand English, 5. singleton pregnancy, 6. low-risk pregnancy, 7. plans to deliver at one of five participating health care settings in Shelby Co, TN. Exclusion criteria: 1. less than 16 years of age or greater than 40 years of age; 2. multiple gestation in the current pregnancy; 3. chronic hypertension or vascular disease requiring therapy; 4. maternal red cell alloimmunization, except Rh factor; 5. hemoglobinopathy, including sickle cell trait and severe iron deficiency anemia (hemoglobin <9); 6. insulin-dependent diabetes; 7. appreciable renal or cardiopulmonary disease. 8. prolapsed or ruptured membranes; 9. oligohydramnios; 10. complete placenta previa; 11. refused consent, endocrine disease, collagen disease, active or chronic hepatitis, renal disease, pulmonary or heart disease requiring therapeutic medication or limitation of physical activity (except for mitral valve prolapse or asthma requiring only occasional medication), major fetal anomaly, infection with HIV, delivery or prenatal care outside clinical center.	We examined early-life predictors of child socioemotional and neurocognitive development, including the CBCL.
K	The ECHO PATHWAYS cohort included pregnant women and children delivered at three Washington hospitals serving diverse populations. Participants contributed survey data and specimens to the Global Alliance to Prevent Prematurity and Stillbirth (GAPPS) biorepository during their pregnancy (from 2012 to 2017). Inclusion criteria: 1. pregnant women 14 years of age, 2. women 18 years of age who are no longer pregnant but have delivered within 7 days of enrollment. Exclusion criteria: 1. under 18 years of age and not medically or legally emancipated, without parental consent, 2. anyone unable to provide informed consent, 3. received narcotics in the 12 hours prior to being asked to provide consent, 4. in active labor by their physician's standards, 5. in preterm labor 21 0/7 weeks gestation, 6. multiple gestation pregnancy.	The GAPPS established a data and tissue bio-bank to be used to examine normal and abnormal pregnancies, including how pregnancy affects maternal and child health and neurodevelopmental functioning, including the CBCL at ages 4-6 and 8 years.

© 2023 Hofheimer JA et al. *JAMA Network Open.*

Cohort	Sampling, inclusion, and exclusion criteria	Study aims and assessments
L	The Neonatal Neurobehavior and Outcomes in Very Preterm Infants (NOVI) Study enrolled 704 infants from nine university-affiliated NICUs in Rhode Island, Mississippi, Missouri, Hawaii, North Carolina, and California from April 2014 through June 2016. Inclusion criteria: 1. <30 weeks postmenstrual age (PMA), 2. parental ability to read and speak English (two sites allow Spanish speaking), 3. reside within 3 hours of the NICU and follow-up clinic. Exclusion criteria: 1. major congenital anomalies affecting NICU Network Neurobehavioral Scale (NNNS) administration prior to discharge; 2. maternal age <18 years, cognitive impairment, or death.	To determine which infants born at <30 weeks gestation are at greatest risk for impaired development based on the NNNS and medical risk score and the role of the postnatal environment in moderating risks on early childhood developmental outcomes. Follow-up child assessments yearly at 2 through 6 years of age included the CBCL and other cognitive, language, motor, and executive function assessments, with diagnostic measures of cerebral palsy and autism spectrum disorders.
M	The Early Growth and Development Study (EGDS; N=361) is a longitudinal prospective adoption study of biological parents, adoptive parents, and adopted children enrolled across the United States through research teams based in California; Minnesota; Washington, D.C.; Oregon; and Pennsylvania. The sub-sample for the present study included prenatal data from the biological mother and postnatal maternal and environmental data from the adoptive/rearing mother (since birth). Inclusion criteria: 1. domestic adoption placement, 2. placement occurred within 3 months postpartum, 3. the infant was placed with an adoptive family that was not biologically related to the child, 4. birth and adoptive parents were able to understand English at the 8th-grade level, 5. birth and adoptive family both separately agreed to participate. Exclusion criteria: 1. any known major medical conditions, such as extreme prematurity or extensive medical surgeries.	Children were followed from age 9 months to 15 years to examine associations between family environment and genetics with child neurodevelopment, including measures of temperament, family interaction, behavior (CBCL), positive health, and obesity.
N	Cohort II of EGDS (N=200) is the second longitudinal prospective adoption study of biological parents, adoptive parents, and adopted children enrolled across the United States through research teams based in California; Minnesota; Washington, D.C.; Oregon; and Pennsylvania . The sub-sample for the present study included data from the adoptive/rearing mother (since birth) at the time of the CBCL assessments and prenatal data from the biological mother. Inclusion criteria: 1. domestic adoption placement, 2. placement occurred within 3 months postpartum, 3. the infant was placed with an adoptive family that was not biologically related to the child, 4. birth and adoptive parents were able to understand English at the 8th-grade level, 5. birth and adoptive family both separately agreed to participate. Exclusion criteria: 1. any known major medical conditions, such as extreme prematurity or extensive medical surgeries.	Children were followed from age 9 months to 15 years in ECHO to examine associations between family environment and genetics associated with child neurodevelopment, including measures of temperament, family interaction, behavior (CBCL), positive health, and obesity.

© 2023 Hofheimer JA et al. *JAMA Network Open.*

Cohort	Sampling, inclusion, and exclusion criteria	Study aims and assessments
O	The Magee cohort follows Pittsburgh women oversampled for childhood trauma from the 1st trimester. The Rochester cohort study, which oversamples for psychosocial stress, follows a sample of women from the 1st trimester.<br>Inclusion criteria: 1. 18-40 years of age, 2. <13 weeks gestation at start, 3. medically normal risk, singleton pregnancy, 4. no history of psychotic illness, 5. ability to communicate in English. Exclusion criteria: 1. presence of significant immunological, endocrinological or other significant medical condition; 2. <18 years of age.	Aims are to examine if and how prenatal exposures "program" adaptive biological responses in the fetus and child and carry-forward effects on neurodevelopmental and behavioral (CBCL) health and obesity, with a focus on inflammation as a biological mechanistic pathway.
P	Prospective pregnancy cohort, enrolling women at first prenatal visit; convenience sample enrolled 2008-2016 from three prenatal clinics in Lansing, Michigan; N=516 mothers and 535 children. Inclusion criteria: 1. over age 18, 2. able to be interviewed in English, 3. making their first prenatal visit, 4. provider has introduced project to potential subject and she has agreed to talk to our recruiter.	Study collected prospective prenatal interviews and biological samples with follow-up to examine pre- and perinatal environmental contaminants, nutritional factors, and inflammation associated with childhood illnesses and obesity, and neurodevelopmental and behavioral outcomes, including CBCL assessments.
Q	This is a prospective prebirth cohort study begun through the Children's Environmental Health Research Center at Illinois. Recruitment of pregnant women started in late 2013 and is still ongoing, with the goal of recruiting 720 women in the first trimester of pregnancy and following 600 infants prospectively from birth to 4 years. Inclusion criteria: 1. pregnant women must be between 18-40 years of age, 2. fluent in English, 3. live within 30-minute drive to research lab; 4. prenatal care must be done at two specific clinics in Champaign-Urbana area, 5. birth of infant must happen at Presence Medical Center or Carle Foundation Hospital in Urbana, 6. only one child per mother may participate, 7. able to enroll in study and provide first urine sample by 14 weeks gestation, 8. able/willing to come to research lab for child assessments post-delivery. Exclusion criteria: 1. high-risk pregnancy, including expecting multiple births; 2. a serious health condition involving either the child or mother during pregnancy; 3. a health condition that prevents or limits the child's participation in postnatal assessments; 4. mother loses custody of child and/or is unwilling or unable to complete postnatal surveys.	Aims were to examine the unique and combined impact of prenatal exposure to endocrine disrupting chemicals and maternal stress on child neurodevelopmental health and functioning.

© 2023 Hofheimer JA et al. *JAMA Network Open.*

Cohort	Sampling, inclusion, and exclusion criteria	Study aims and assessments
R	Utah population-based nuMoM2b Study began in 2010 and studied nulliparous pregnant women. Inclusion criteria: 1. nullipara pregnant women with no prior pregnancy lasting 20 weeks 0 days or greater; 2. viable singleton gestation, a single living fetus with fetal cardiac activity at the most recent ultrasound before enrollment; 3. between 6 weeks 0 days and 13 weeks 6 days project estimated gestational age (EGA) at first study visit; 4. intend to deliver at a participating hospital.<br>Exclusion criteria: 1. participant age <18 years 2. history of three or more spontaneous abortions; 3. fetal malformation evident at or before enrollment that is likely lethal (e.g., anencephaly, hydrops, diffuse subcutaneous edema or cystic hygroma, ectopic cordis, encephalocele); 4. known fetal aneuploidy (based on chorionic villus sampling); 5. surrogate pregnancy (donor oocyte pregnancy); 6. multifetal reduction; 7. participating in an intervention study that is anticipated to influence maternal or fetal morbidities/mortality unless it is determined before enrollment that the study code will be made available; 8. women previously enrolled in this study, including those consented but delivered before 20 weeks 0 days gestation; 9. planned pregnancy termination; 10. unable to provide informed consent.	This prospective cohort study evaluated the underlying, interrelated mechanisms of several primary outcomes, including preterm birth, preeclampsia, fetal growth restriction, and stillbirth. The study aims to examine the role of genetics and the environment on children's growth, obesity, respiratory health, asthma, and child development, including CBCL outcomes.
S	N=162 pregnant women in Utah. Inclusion criteria:1. pregnant women ages 18-39; 2. singleton pregnancy. Exclusion criteria: 1. maternal health complications (heart disease, asthma, cancer, weight gain in excess of 60 lbs. at start of the 3rd trimester, gestational diabetes, or preeclampsia); 2. inability to communicate in English or Spanish, provide consent, or complete questionnaires.	The objective of the study is to examine physiological and epigenetic mechanisms by which maternal prenatal emotion dysregulation could affect newborn neurobehavioral outcomes and CBCLs at 3 years.
T	The PRISM cohort was originally funded by the National Heart, Lung, and Blood Institute (NHLBI) to support the establishment of a longitudinal pregnancy cohort (N=350). Inclusion criteria: 1. mother 18 years of age or older at recruitment in pregnancy, 2. single gestation pregnancy, 3. mother English- or Spanish-speaking. Exclusion criteria: 1. at enrollment, endorsement of drinking >= 7 alcoholic drinks/week prior to pregnancy recognition, 2. at enrollment, endorsement of any drinking after pregnancy recognition, 3. maternal or child chronic health conditions that would impede study participation.	The PRISM examines the impact of maternal and child pre- and postnatal stress exposures (e.g., air pollution and nutrition) on maternal prenatal and child epigenetic characteristics, stress regulation and on child health outcomes in infancy and early childhood, with a focus on respiratory health. Aims at ages 3 and 5 examine pathways among maternal and child stress exposures and child telomere length, stress reactivity, and neurodevelopment.

© 2023 Hofheimer JA et al. *JAMA Network Open.*

eTable 2. Growth Mixture Modeling Fit Statistics

Title	LL	Parameters	BIC	aBIC	Entropy	LMR value	LMR, p-value	BLRT_KM1LL	BLRT, p-value	min_N	max_N
1 classes	-23417.85	9	46910.20	46881.60						3934	3934
2 classes	-22651.84	12	45403.01	45364.88	0.95	1472.72	.02	-23417.85	0	273	3661
3 classes	-22241.24	15	44606.65	44558.99	0.92	789.40	.02	-22651.84	0	89	3366
4 classes	-21997.40	18	44143.79	44086.60	0.89	468.81	.10	-22241.24	0	64	3160

LL, log likelihood value; BIC, Bayesian information criterion; aBIC, Akaike's Bayesian information criterion; LMR, Lo-Mendell-Rubin statistic; BLRT, bootstrapped likelihood ratio test; BLRT_KM1LL, log likelihood of the K-1 model (one less class) for the bootstrapped likelihood ratio test; max, maximum; min, minimum.

© 2023 Hofheimer JA et al. *JAMA Network Open.*

eTable 3. Prenatal Substance Use Among Mothers with Prior Psychiatric Diagnosis and/or Positive PROMIS Depression Screen

**History of maternal psychiatric disorder up until CBCL administration, N (%) with data**	3,158 (80.3)
Yes, N (%)	699 (22.1)
Yes, among non-exposed group, N (%)	448 (16.1)
Yes, among alcohol group, N (%)	130 (19.4)
Yes, among tobacco group, N (%)	130 (27.2)
Yes, among marijuana group, N (%)	64 (22.1)
Yes, among illicit substances group, N (%)	35 (36.5)
Yes, among opioid group, N (%)	33 (50.0)
**Maternal PROMIS depressive symptom score up until CBCL administration, N (%) with data**	2,451 (62.3)
Mean (min-max, SD)	50 (33-78, 8.3)
Mean among non-exposed group (min-max, SD)	49 (33-78, 8)
Mean among alcohol group (min-max, SD)	52 (33-74, 8.1)
Mean among tobacco group (min-max, SD)	53 (33-76, 9)
Mean among marijuana group (min-max, SD)	52 (33-74, 9.2)
Mean among illicit substances group (min-max, SD)	53 (33-69, 9.8)
Mean among opioid group (min-max, SD)	51 (39-69, 9.7)
T>=55, N (%)	664 (27.1)

CBCL, Child Behavior Checklist; SD, Standard Deviation; min, minimum; max, maximum; PROMIS, Patient-Reported Outcomes Measurement Information System®.

© 2023 Hofheimer JA et al. *JAMA Network Open.*

eTable 4. Distribution of Psychosocial Adversity Index Variables Among Mothers With versus Without a Prior Psychiatric Diagnosis and/or Positive Depressive Symptom Screen

	Prior maternal psychiatric disorder and/or positive depressive symptom screen	No prior maternal psychiatric disorder and/or positive depressive symptom screen	P value
Number of pregnancies	1,212	1,269	
Age at delivery, N(%) with data	1,210 (99.0)	1,264 (99.0)	< 0.001
<21 years	142 (11.7)	139 (11.0)	
>=21 years	1,068 (88.3)	1,125 (89.0)	
Highest level of education, N(%) with data	1,151 (95.0)	1,097 (86.4)	0.04
<= High School, N(%)	228 (19.8)	205 (18.7)	
Some College and above, N(%)	923 (80.2)	892 (81.3)	
Some College, N(%)	393 (42.6)	367 (41.1)	
Bachelor Degree and above, N(%)	530 (57.4)	525 (58.9)	
Marital Status, N(%) with data	622 (51.3)	372 (29.3)	0.007
Married or living with a partner, N(%)	427 (68.6)	266 (71.5)	
Public/no Insurance, N(%) with data	632 (52.1)	1,158 (91.3)	<0.001
Yes, N(%)	441 (69.8)	624 (53.9)	

© 2023 Hofheimer JA et al. *JAMA Network Open.*
