# Supplementary material for: Assessment of Psychosocial and Neonatal Risk Factors for Trajectories of Behavioral Dysregulation Among Young Children From 18 to 72 Months of Age
Source: JAMA Netw Open. 2023 Apr 26;6(4):e2310059. doi: 10.1001/jamanetworkopen.2023.10059 (PMC10134008; doi:10.1001/jamanetworkopen.2023.10059)
Supplement: Supplement 2. — Nonauthor Collaborators [file jamanetwopen-e2310059-s002.pdf]

*Indicates required information. Only first name, last name, and suffix will appear in PubMed.

*Group Name(s): The Program Collaborators for Environmental influences on Child Health Outcomes							
***First Name and Middle Initial(s)**	***Last Name**	***Suffix (eg, Jr, III)**	Academic Degrees	Institution	Location (city, state/province, country)	Role or Contribution, eg, chair, principal investigator	Group (if more than 1 Group listed in the byline) and/or Subgroup (eg, Steering Committee)
Phillip B	Smith		MD	Duke Clinical Research Institute	Durham, North Carolina, USA	ECHO Coordinating Center Prinicipal Investigator	ECHO Coordinating Center U2COD023375
Laura K	Newby		MD	Duke Clinical Research Institute	Durham, North Carolina, USA	ECHO Coordinating Center Prinicipal Investigator	ECHO Coordinating Center U2COD023375
Lisa P	Jacobson		PhD	Johns Hopkins University	Baltimore, Maryland, USA	ECHO Data Analysis Center Principal Investigator	ECHO Data Analysis Center U24D023382
Diane J	Catellier		PhD	Research Triangle Park Institute	Durham, North Carolina, USA	ECHO Data Analysis Center Principal Investigator	ECHO Data Analysis Center U24D023382
Richard C	Gershon		PhD	Northwestern University School of Medicine	Evanston, Illinois, USA	ECHO Person Reported Outcome Core Principal Investigator	ECHO Person Reported Outcome Core U24OD023319
David	Cella		PhD	Northwestern University School of Medicine	Evanston, Illinois, USA	ECHO Person Reported Outcome Core Principal Investigator	ECHO Person Reported Outcome Core U24OD023319
Susan L	Teitelbaum		PhD	Icahn School of Medicine at Mount Sinai	New York, NY, USA	ECHO Cohort Principal Investigator	ECHO Cohort UH3OD023320
Annemarie	Stroustrup		MD	Icahn School of Medicine at Mount Sinai	New York, NY, USA	ECHO Cohort Principal Investigator	ECHO Cohort UH3OD023320
Andrea L	Lampland		MD	Children’s Hospital and Clinic	Minneapolis, MN, USA	ECHO Cohort Principal Investigator	ECHO Cohort UH3OD023320
Mark L	Hudak		MD	University of Florida College of Medicine	Jacksonville, FL, USA	ECHO Cohort Principal Investigator	ECHO Cohort UH3OD023320

*Indicates required information. Only first name, last name, and suffix will appear in PubMed.

***First Name and Middle Initial(s)**	***Last Name**	***Suffix (eg, Jr, III)**	Academic Degrees	Institution	Location (city, state/province, country)	Role or Contribution, eg, chair, principal investigator	Group (if more than 1 Group listed in the byline) and/or Subgroup (eg, Steering Committee)
Dennis E	Mayock		MD	University of Washington	Seattle, WA, USA	ECHO Cohort Principal Investigator	ECHO Cohort UH3OD023320
Lisa K	Washburn		MD	Wake Forest University School of Medicine	Winston Salem, NC	ECHO Cohort Principal Investigator	ECHO Cohort UH3OD023320
Cristiane	Duarte		PhD	New York State Psychiatric Institute	New York, NY, USA	ECHO Cohort Principal Investigator	ECHO Cohort UH3OD023328
Glorisa J	Canino		PhD	University of Puerto Rico	San Jaun, PR,	ECHO Cohort Principal Investigator	ECHO Cohort UH3OD023328
Assiamira M	Ferrara		MD	Kaiser Permanente Northern California Division of Research	Oakland, CA, USA	ECHO Cohort Principal Investigator	ECHO Cohort UH3OD023289
Caherine J	Karr		MD	University of Washington, Department of Environmental and Occupational Health Sciences	Seattle, WA, USA	ECHO Cohort Principal Investigator	ECHO Cohort UH3OD023271
Alex	Mason		PhD	University of Tennessee Health Science Center	Memphis, TN, USA	ECHO Cohort Principal Investigator	ECHO Cohort UH3OD023271
Carmen J	Marsit		PhD	Emory University	Atlanta, GA, USA	ECHO Cohort Principal Investigator	ECHO Cohort UH3OD023347
Steven L	Pastyrnak		PhD	Helen DeVos Children's Hospital	Grand Rapids, MI, USA	ECHO Cohort Principal Investigator	ECHO Cohort UH3OD023347
Charles	Neal		MD	Kapiolani Medical Center for Women and Children	Providence, RI, USA	ECHO Cohort Principal Investigator	ECHO Cohort UH3OD023347
Brian S	Carter		MD	Children's Mercy	Kansas City, MO, USA	ECHO Cohort Principal Investigator	ECHO Cohort UH3OD023347
Jennifer B	Helderman		MD	Wake Forest University School of Medicine	Winston Salem, NC	ECHO Cohort Principal Investigator	ECHO Cohort UH3OD023347
Jody M	Ganiban		PhD	George Washington University	Washington, DC	ECHO Cohort Principal Investigator	ECHO Cohort UH3OD023389
Thomas G	O'Connor		PhD	University of Rochester Medical Center Rochester	New York, NY, USA	ECHO Cohort Principal Investigator	ECHO Cohort UH3OD023349
Hyagriv	Simhan		MD	University of Pittsburgh Medical Center, Magee Women's Hospital	Pittsburgh, PA, USA	ECHO Cohort Principal Investigator	ECHO Cohort UH3OD023349

*Indicates required information. Only first name, last name, and suffix will appear in PubMed.

***First Name and Middle Initial(s)**	***Last Name**	***Suffix (eg, Jr, III)**	Academic Degrees	Institution	Location (city, state/province, country)	Role or Contribution, eg, chair, principal investigator	Group (if more than 1 Group listed in the byline) and/or Subgroup (eg, Steering Committee)
Jean	Kerver		PhD	Michigan State University	East Lansing, MI, USA	ECHO Cohort Principal Investigator	ECHO Cohort UH3OD023285
Charles	Barone		MD	Henry Ford Health System	Detroit, MI, USA	ECHO Cohort Principal Investigator	ECHO Cohort UH3OD023285
Patricia	McKane		DVM	Michigan Department of Health and Human Services	Lansing, MI, USA	ECHO Cohort Principal Investigator	ECHO Cohort UH3OD023285
Nigel	Paneth		MD	Michigan State University	East Lansing, MI, USA	ECHO Cohort Principal Investigator	ECHO Cohort UH3OD023285
Michael R	Elliott		PhD	University of Michigan	Ann Arbor, MI, USA	ECHO Cohort Principal Investigator	ECHO Cohort UH3OD023285
Susan L	Schantz		PhD	University of Illinois, Beckman Institute	Urbana, IL, USA	ECHO Cohort Principal Investigator	ECHO Cohort UH3OD023272
Robert M	Silver		MD	University of Utah	Salt Lake City, UT, USA	ECHO Cohort Principal Investigator	ECHO Cohort UH3OD023249
Rosalind J	Wright		MD	Icahn School of Medicine at Mount Sinai	New York, NY, USA	ECHO Cohort Principal Investigator	ECHO Cohort UH3OD023337
Michelle	Bosquet-Enlow		PhD	Boston Children’s Hospital	Boston, MA, USA	ECHO Cohort Principal Investigator	ECHO Cohort UH3OD023337
Joanna A	Maselko		ScD	University of North Carolina Gillings School of Global Public Health	Chapel Hill, NC, USA	Consultant: Psychiatric epidemiology	ECHO Cohort UH3ODO23348
