# Supplementary material for: Assessment of Psychosocial and Neonatal Risk Factors for Trajectories of Behavioral Dysregulation Among Young Children From 18 to 72 Months of Age
Source: JAMA Netw Open. 2023 Apr 26;6(4):e2310059. doi: 10.1001/jamanetworkopen.2023.10059 (PMC10134008; doi:10.1001/jamanetworkopen.2023.10059)
Supplement: Supplement 3. — Data Sharing Statement [file jamanetwopen-e2310059-s003.pdf]

Data Sharing Statement

Hofheimer. Assessment of Psychosocial and Neonatal Risk Factors for Trajectories of Behavioral Dysregulation Among Young Children From 18 to 72 Months of Age. *JAMA Netw Open*. Published April 26, 2023. doi:10.1001/jamanetworkopen.2023.10059

Data
Data available: Yes
Data types: Deidentified participant data
How to access data: De-identified data from the ECHO Program are available through NICHD's Data and Specimen Hub (DASH). DASH is a centralized resource that allows researchers to access data from various studies via a controlled-access mechanism. Researchers can now request access to these data by creating a DASH account and submitting a Data Request Form. The NICHD DASH Data Access Committee will review the request and provide a response in approximately two to three weeks. Once granted access, researchers will be able to use the data for three years. See the DASH Tutorial for more detailed information on the process.
When available: With publication

Supporting Documents
Document types: None

Additional Information
Who can access the data: TBD by NICHD's Data and Specimen Hub (DASH)
Types of analyses: TBD by NICHD's Data and Specimen Hub (DASH)
Mechanisms of data availability: TBD by NICHD's Data and Specimen Hub (DASH)
